# Human plasmacytoid dendritic cells at the crossroad of type I interferon-regulated B cell differentiation and antiviral response to tick-borne encephalitis virus

**DOI:** 10.1371/journal.ppat.1009505

**Published:** 2021-04-15

**Authors:** Marilena P. Etna, Aurora Signorazzi, Daniela Ricci, Martina Severa, Fabiana Rizzo, Elena Giacomini, Andrea Gaggioli, Isabelle Bekeredjian-Ding, Anke Huckriede, Eliana M. Coccia

**Affiliations:** 1 Department of Infectious Diseases, Istituto Superiore di Sanità, Rome, Italy; 2 Department of Medical Microbiology & Infection Prevention, University of Groningen, Groningen, The Netherlands; 3 National Center for the Control and Evaluation of Medicines, Istituto Superiore di Sanità, Rome, Italy; 4 Division of Microbiology, Paul-Ehrlich-Institut, Langen, Germany; Emory University, UNITED STATES

## Abstract

The Tick-borne encephalitis virus (TBEV) causes different disease symptoms varying from asymptomatic infection to severe encephalitis and meningitis suggesting a crucial role of the human host immune system in determining the fate of the infection. There is a need to understand the mechanisms underpinning TBEV-host interactions leading to protective immunity. To this aim, we studied the response of human peripheral blood mononuclear cells (PBMC) to the whole formaldehyde inactivated TBEV (I-TBEV), the drug substance of Encepur, one of the five commercially available vaccine. Immunophenotyping, transcriptome and cytokine profiling of PBMC revealed that I-TBEV generates differentiation of a sub-population of plasmacytoid dendritic cells (pDC) that is specialized in type I interferon (IFN) production. In contrast, likely due to the presence of aluminum hydroxide, Encepur vaccine was a poor pDC stimulus. We demonstrated I-TBEV-induced type I IFN together with Interleukin 6 and BAFF to be critical for B cell differentiation to plasmablasts as measured by immunophenotyping and immunoglobulin production. Robust type I IFN secretion was induced by pDC with the concerted action of both viral E glycoprotein and RNA mirroring previous data on dual stimulation of pDC by both *S*. *aureus* and influenza virus protein and nucleic acid that leads to a type I IFN-mediated sustained immune response. E glycoprotein neutralization or high temperature denaturation and inhibition of Toll-like receptor 7 signalling confirmed the importance of preserving the functional integrity of these key viral molecules during the inactivation procedure and manufacturing process to produce a vaccine able to stimulate strong immune responses.

## Author summary

Though vaccination is generally considered effective in reducing tick-borne encephalitis (TBE) incidence, several studies have shown that the antibody response to TBEV vaccination declines with age resulting in more frequent TBE cases among 50+ year-old vaccinees. These observations together with the lack of a specific antiviral drug impose to pinpoint novel host- and pathogen-directed therapies and to improve the control of vaccine efficacy. Thus, we interrogated *in vitro* human PBMC, whose response to TBEV may provide a picture closer to what occurs *in vivo* in humans after vaccination or natural infection compared to animal models. The role of E glycoprotein and viral RNA in promoting antiviral and B cell-mediated responses was investigated. Thus, these key viral molecules should be considered, in future, for novel subunit vaccine formulations than the current whole inactivated TBEV-based vaccines, which require laborious manipulation in biosafety level-3 laboratory and animal testing for manufacturing and batch release.

## Introduction

Tick-borne encephalitis (TBE) is a severe neurological disease caused by tick-borne encephalitis virus (TBEV), a single-stranded, positive-sense RNA virus, belonging to the Flaviviridae family. Being poorly adapted to humans, TBEV can lead to a variety of clinical manifestations ranging from mild fever to severe neurological illness with a high risk for long-lasting sequelae and with high mortality rate [1]. Endemic throughout the forested area of Europe and Asia, TBEV is considered an emerging pathogen because of its expansion into new geographical regions and increased incidence of human infections [2]. TBEV is a zoonotic agent whose transmission cycle involves the *Ixodes* tick, that acts as both vector and reservoir, and small mammals (rodents or shrews) as reservoir and amplifying host, while humans are accidentally infected. Taxonomically, three subtypes of TBEV exist: the European (TBEV-Eu), the Far-eastern (TBEV-FE) and the Siberian (TBEV-Sib) subtypes, which are all closely related both genetically and antigenically [3].

Currently, there is no specific treatment for TBE. Thus, it is extremely important to prevent infection by vaccination [4]. Five licensed vaccines have been developed by formalin-inactivation of different TBEV subtypes. In particular, two European vaccines have been produced to address the clinical need: FSME-IMMUN (Pfizer, USA), prepared from the Neudoerfl strain of the European subtype [5], and Encepur (GSK), based on the Karlsruhe (K23) strain [6,7]. These vaccines have been used for more than 30 years and are highly effective in preventing TBE although a booster vaccination is foreseen after 3–5 years [5].

Despite TBE clinical importance, many aspects of TBEV infection and pathogenesis remain unclear. Therefore, investigating the human immune response to this infection is important to gain further understanding into treatment and prevention of the disease. As for other viral families, in the case of several flaviviruses, the innate host defense mostly depends on the antiviral activity of a heterogeneous group of cellular antiviral proteins that include virus sensors and intra- and extracellular signal mediators such as type I interferon (IFN) [8]. Indeed, the IFN response is a fundamental part of the innate immune system due to its capacity to activate the expression of several genes, known as IFN-stimulated genes (ISGs), which in turn inhibit virus multiplication at the level of transcription, translation, genome replication, assembly and exit and stimulate subsequent adaptive immune response [9,10]. The importance of this pathway for controlling flavivirus replication has been also addressed by Lindqvist et al. in the CNS, where astrocytes mount a rapid IFN response to restrict viral spread [11]. In addition, several studies further sustained the relevance of the IFN pathway by showing that in different populations predisposition to severe forms of TBE and susceptibility to TBEV-induced disease are associated with single nucleotide polymorphism and intronic polymorphism in IFN and ISGs [12–14]. Notwithstanding, for TBEV, the antiviral type I IFN response is not well characterized in humans although this virus was used as model of infection in the first IFN studies [15].

In the present study we focused on the interaction of human immune cells with the TBEV vaccine, Encepur, as well as with the single components present in the final formulation of the product, including inactivated non-replicating TBEV (I-TBEV). In particular, human peripheral blood mononuclear cells (PBMC) and, alternatively, purified plasmacytoid dendritic cells (pDC), main producers of type I IFN [16], were used to analyze both the innate and adaptive immune response against TBEV in an *in vitro* setting. In addition, the impact of viral RNA as well as TBEV E glycoprotein was studied to evaluate the contribution of these viral pathogen-associated molecular patterns to PBMC stimulation. Although this study was entirely conducted *in vitro*, our data shed light on the crosstalk between pDC and B cells in mounting an anti-TBEV response in a type I IFN-dependent manner. In conclusion, our findings reveal a key role for TBEV molecules, namely E glycoprotein and viral RNA, in activating pDC and, in turn, in establishing the antiviral immune response.

## Results

### ENCEPUR vaccine and I-TBEV differentially promote cytokine and chemokine production in human PBMC

**Fig 1:**
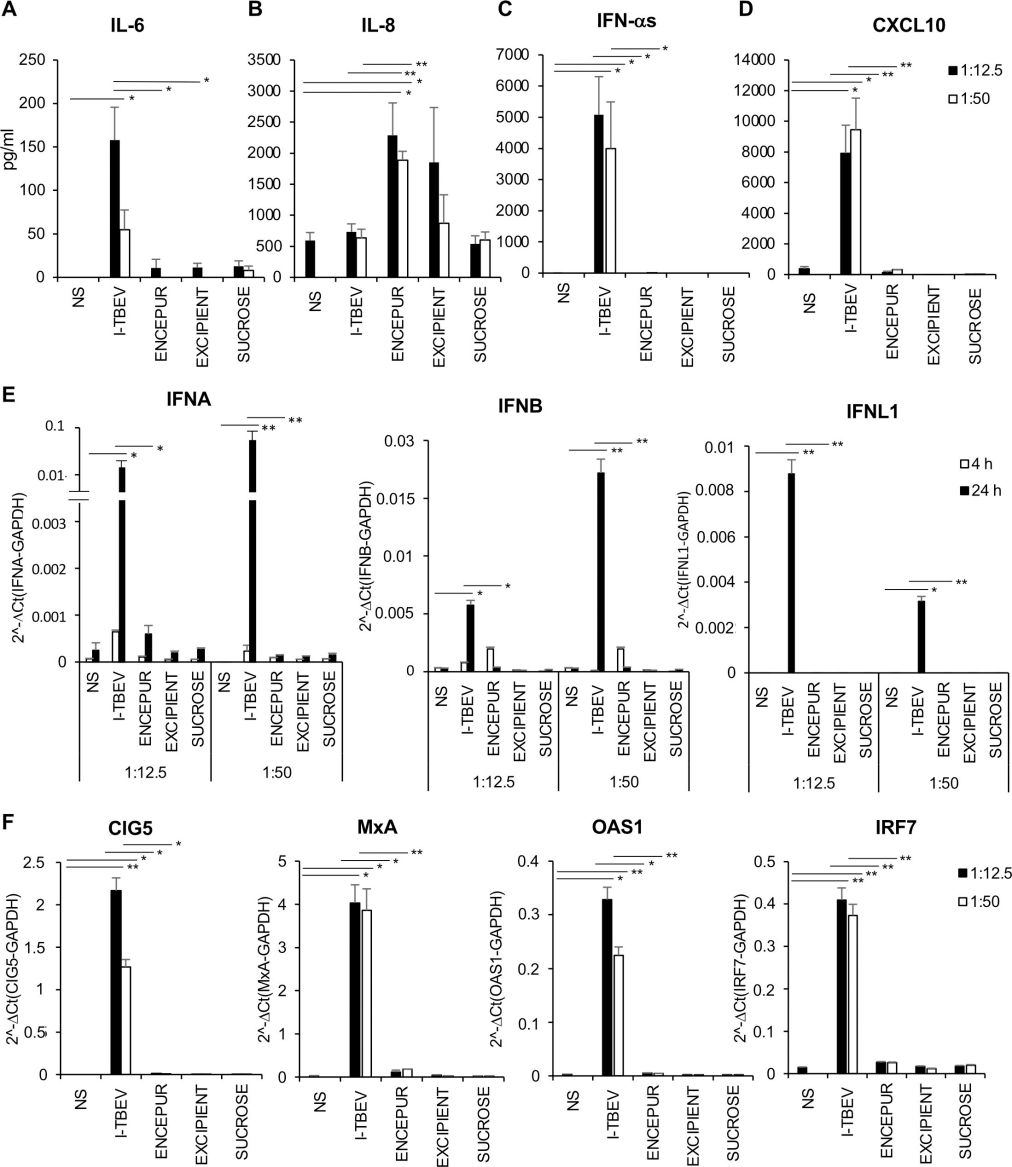
Cytokine and chemokine release in PBMC cultures stimulated with Encepur and single vaccine components. PBMC were stimulated for 4 and 24 hours with inactivated TBEV (I-TBEV), Encepur vaccine, excipient and sucrose (dilution 1:12.5 and 1:50) or left untreated (NS). The production of IL-6 (A), IL-8 (B), IFN-α (C) and CXCL10 (D) was tested by cytometric bead assay or ELISA in 24 hour-collected supernatants. The results shown were mean values ± SEM of 6 independent experiments. ANOVA p value for IL-6: 0.004; for IL-8: 0.021; for IFN-α: 0.001; for CXCL10: 0.001. Sidak adjustment for multiple comparisons was applied to IL-6, IFN-α and CXCL10, while LSD (equivalent to no adjustments) was used for IL-8. (E-F) Relative expression of IFNA, IFNB, IFNL1 (at 4 hours, grey bars and 24 hours, black bars) and of CIG5, MxA, OAS1 and IRF7 (at 24 hours) in PBMC left untreated (NS) or stimulated with I-TBEV, Encepur vaccine, excipient and sucrose (dilution 1:12.5 and 1:50) was measured by q-PCR analysis. All quantification data are normalized to GAPDH level by using the equation 2^−ΔCt^. The results shown were mean relative values ± SEM of 4 independent experiments. ANOVA p value for IFNA, IFNB, IFNL1, CIG5 and MxA: 0.000; for OAS1: 0.002; for IRF7: 0.001. Sidak adjustment for multiple comparisons was applied for the analysis of all target genes.

Reasoning that cytokines and chemokines produced by innate immune cells play a key role in first line defence against viruses, we investigated whether Encepur vaccine or its components would be able to stimulate in PBMC the secretion of these soluble immune mediators, namely interleukin-6 (IL-6), IL-8, IFN-α and C-X-C Motif Ligand 10 (CXCL10) (**Fig 1**). Of note, the TBE vaccination status of the blood donors employed in the present study was unknown; however, given the absence of national recommendation and the low incidence of TBE in the Italy (with a total of only 16 cases in 2020), it is highly unlikely that the donors had been previously exposed to the vaccine or the virus [17]. Encepur is composed of the non-replicating inactivated TBEV (I-TBEV) adsorbed on aluminum hydroxide in presence of sucrose as stabilizer. Accordingly, we compared the effects induced on PBMC by Encepur and the single components, namely I-TBEV, excipient containing aluminum hydroxide and sucrose. After 24 hours of PBMC stimulation, I-TBEV promoted high levels of IL-6, IFN-α and CXCL-10 while Encepur stimulated only IL-8 secretion to a similar extent as the excipient, thus suggesting that the latter is the major inducer (**Fig 1A–1D**). No significant modulations in cytokine and chemokine release were found in supernatants of sucrose-stimulated PBMC.

To understand whether this profile might be related to differences in cell viability, PBMC were stained by FvDye to evaluate the percentage of the dead cells by flow cytometric analysis (**S1 Table**). Although cell death was slightly higher in PBMC treated for 24 hours with Encepur and excipient matrix compared to I-TBEV and sucrose, the majority of cells remained viable in all conditions, thus indicating that differences in cytokine and chemokine profile cannot arise from cell viability.

Given the well-characterized interference of aluminum hydroxide with the detection of soluble proteins [18], experiments were planned to evaluate whether this occurs in our experimental system. To this aim, the potential interfering effect of the excipient matrix was investigated by ELISA on IFN-α production, whose expression mirrored those of CXCL-10 and IL-6. Having found that no technical interference was observed (**S2A Table**) when excipient was also added to the IFN-α ELISA standard, then we investigated whether a biological interference might occur during the *in vitro* stimulation of PBMC with I-TBEV. Interestingly, when I-TBEV was used in combination with either Encepur or the excipient matrix, type I IFN release was strongly affected (**S2B Table**). In addition, to investigate whether aluminum hydroxide could also interfere at transcriptional level we also studied gene expression of IFNA in the aforementioned conditions (**Fig 1E**). Q-PCR analysis shows that Encepur stimulated only a modest IFNA expression after 24 hours while a robust transcription was observed with I-TBEV starting after 4 hours and increasing at 24 hours (**Fig 1E**). A similar expression profile was also found for IFNB, while IFNL1 showed slower kinetics of induction being transcribed only at 24 hours (**Fig 1E**). The expression of canonical ISGs, such as MxA and IRF-7, and others previously shown to be involved in anti-TBEV response, as CIG5 (Viperin) and OAS1 [9,19,20], was then analyzed (**Fig 1F**). In line with IFN data, all the studied ISGs were induced by I-TBEV only (**Fig 1F**). In the attempt to detect low copy numbers of MxA mRNA possibly induced by Encepur, highly sensitive digital PCR approach was applied. Also in this case, yet, no MxA expression could be detected (**S1 Fig**).

### *In vitro* B cell differentiation and Ig production promoted by I-TBEV was lost in response to ENCEPUR stimulation

**Fig 2:**
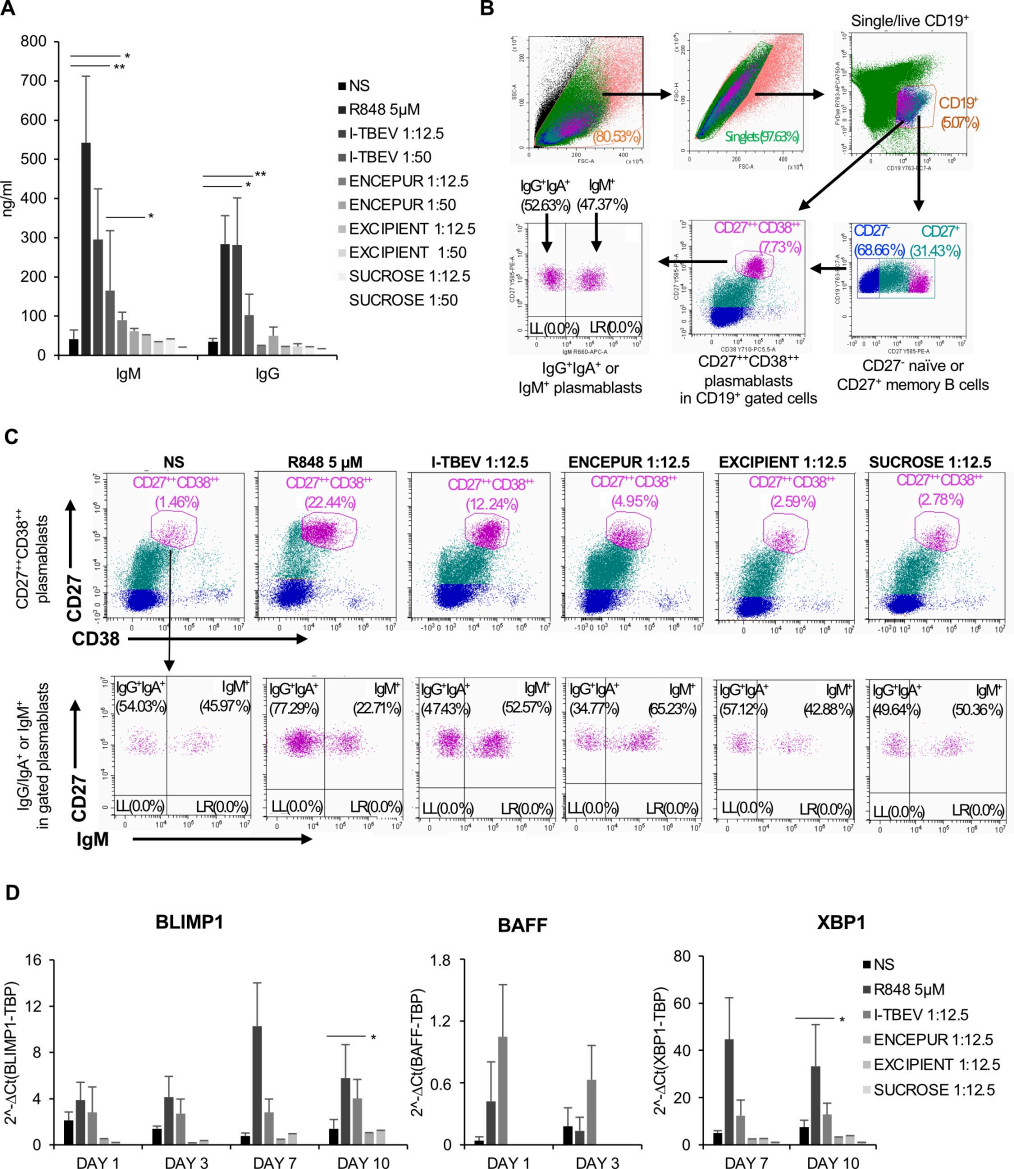
Ig production and B cell differentiation after PBMC stimulation with Encepur and the single vaccine components. (A) PBMC were stimulated with R848 (5 μM), I-TBEV, Encepur vaccine, excipient and sucrose (dilution 1:12.5 and 1:50) or left untreated (NS). The production of total IgM and IgG was measured by ELISA in supernatants of PBMC cultures stimulated for 10 days. The results shown were mean values ± SEM of 6 independent experiments for IgM and 4 independent experiments for IgG. ANOVA p value for IgM: 0.001; for IgG: 0.015. Sidak adjustment for multiple comparisons was applied to IgM while, LSD (equivalent to no adjustments) was used for IgG. (B) Representative dot plots of flow cytometry gating strategy. PBMC were firstly gated by forward (FSC) and side scatter (SSC) then CD19^+^ cells were further gated on live cells. Within the B cell population, plasmablasts were identified as CD27^++^CD38^++^ expressing IgM or IgG/IgA. (C) The percentage of CD27^++^CD38^++^ plasmablasts and IgM^+^ or IgG^+^IgA^+^ plasmablasts was assessed by cytofluorimetric analysis of PBMC stimulated for 10 days with R848 (5 μM), I-TBEV, Encepur vaccine, excipient and sucrose (dilution 1:12.5) or left untreated (NS) as described in B. A representative experiment, out of 5 independent experiments performed, is shown. Numbers in the dot plots are the percentage of live-gated cells positive for CD19^+^CD27^++^CD38^++^ (upper panels). In the lower panels, the percentage of IgM^+^ or IgG^+^IgA^+^ plasmablasts is shown. (D) Relative expression of BLIMP1, BAFF and XBP1 in PBMC left untreated (NS) or stimulated with R848 (5μM), I-TBEV, Encepur vaccine, excipient and sucrose (dilution 1:12.5) for 1, 3, 7 and 10 days was measured by q-PCR analysis. All quantification data are normalized to TBP level by using the equation 2^−ΔCt^. The results shown were mean relative values ± SEM of 3 independent experiments. Student’s t-test p-value for BLIMP1: *p = 0.03. Student’s t-test p-value for XBP-1: *p = 0.03.

Acknowledging that an efficient viral vaccine should promote a robust antibody (Ab) response that specifically recognizes and neutralizes pathogens, we evaluated B cell differentiation in our PBMC-based *in vitro* setting after a 10-day stimulation with Encepur and its components (**Fig 2A**). The results showed that a dose-dependent increase in total immunoglobulin M (IgM) and IgG production was detected in I-TBEV-stimulated PBMC while only low release was found in response to Encepur and, as expected, to excipient and sucrose. In line with these data, flow cytometric analysis revealed that I-TBEV significantly increased the differentiation of both IgM^+^ and IgG^+^IgA^+^ producing CD19^+^CD27^++^CD38^++^ plasmablasts, while only a faint stimulation occurred in response to Encepur (**Fig 2B** and **2C** and **Table 1**). Interestingly, when I-TBEV was used either in combination with Encepur or the excipient matrix, the release of Ig, in particular IgM, was lowered, thus indicating a biological interference of aluminum hydroxide in the PBMC experimental setting (**S2 Fig**).

**Table 1:** Analysis of plasmablast percentage after PBMC stimulation with Encepur vaccine and the different vaccine components.

Experimental Conditions	Plasmablasts (%)	IgM^+^ Plasmablasts (%)	IgG/IgA^+^ Plasmablasts (%)
NS	1.13 ± 0.32	47.25 ± 1.28	52.8 ± 1.23
R848 5μM	15.96 ± 6.49	26.75 ± 4.05	73.25 ± 4.05
I-TBEV 1:12.5	9.98 ± 2.26*	54 ± 1.43*	45.99 ± 1.42*
ENCEPUR 1:12.5	4.02 ± 0.93*	62.43 ± 2.80	37.56 ± 2.80
EXCIPIENT 1:12.5	5.47 ± 2.89	41.72 ± 1.16	58.28 ± 1.16
SUCROSE 1:12.5	3.07 ± 0.29*	47.03 ± 3.33	52.97 ± 3.33

Peripheral blood mononuclear cells (PBMC) were stimulated with R848 (5μM), inactivated TBEV (I-TBEV), Encepur vaccine, excipient and sucrose (1:12.5) or left untreated (NS) for 10 days and the percentages of CD27^hi^CD38^hi^ plasmablasts and IgM^+^ or IgG/IgA^+^ plasmablasts were measured by cytofluorimetric analysis. The results shown were mean relative values ± standard error of the mean of 5 independent experiments. ANOVA p value for plasmablast percentage: 0.018. ANOVA p value for IgM^+^ plasmablast percentage: 0.022. ANOVA p value for IgA/IgG^+^ plasmablast percentage: 0.031. Based on LSD (equivalent to no adjustments).

Subsequently, the expression of BAFF, a cytokine contributing to B cell proliferation and differentiation, and of the two transcription factors BLIMP1 and XBP1, also playing a role in plasma cell differentiation, was analyzed following 1, 3, 7 and 10 days of stimulation. Different kinetics of expression were observed with BAFF mRNA being detectable only in 1- and 3-day I-TBEV stimulated PBMC cultures and BLIMP1 and XBP1 being expressed only at 7 and 10 days. In contrast, no modulation of gene expression was induced by Encepur, excipient matrix or sucrose. R848, a TLR7/8 ligand used as positive control of TLR-driven B cell differentiation, showed a similar expression pattern as induced by I-TBEV (**Fig 2D**).

### TLR7 triggering is involved in pDC activation and type I IFN release by I-TBEV

**Fig 3:**
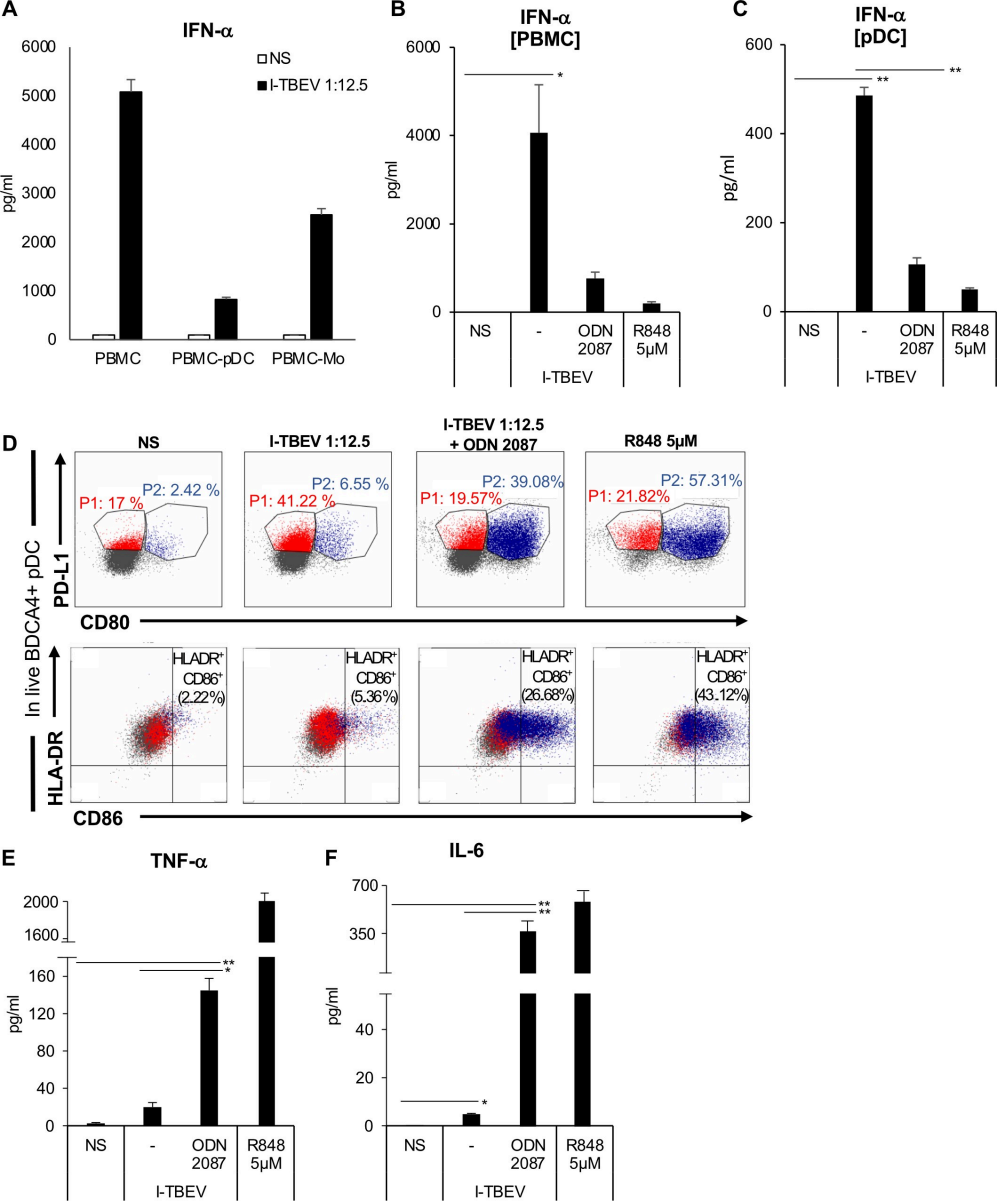
Identification and characterization of cell population responsible for type I IFN production in PBMC. (A) Total PBMC or PBMC depleted of either pDC (PBMC-pDC) or monocytes (PBMC-Mo) were left untreated or stimulated with I-TBEV for 24 hours. The production of IFN-α was measured in culture supernatants by ELISA. The results shown were mean values ± SEM of 3 independent experiments. (B) PBMC were left untreated (NS) or stimulated with R848 (5 μM) or treated with I-TBEV (1:12.5) alone or in combination with the TLR-7/8 inhibitor, ODN 2087. The production of IFN-α was measured by ELISA in supernatants collected after 24 hours. The results shown were mean values ± SEM of 3 independent experiments. ANOVA p value for IFN-α: 0.049. Based on LSD (equivalent to no adjustments). (C-F) Isolated pDC were left untreated (NS) or stimulated for 24 hours with I-TBEV alone or in combination with the TLR-7/8 inhibitor, ODN 2087. The production of IFN-α (C) was measured in culture supernatants by ELISA. The results shown are mean values ± SEM of 3 independent experiments. ANOVA p value for IFN-α: 0.000. Based on LSD (equivalent to no adjustments). (D) Isolated pDC were stained with PD-L1, HLA-DR, CD86, CD80, ILT7, BDCA2 and BDCA4. A total of 50.000 cells were analyzed per sample by flow cytometry to evaluate the percentage of pDC sub-populations in live BDCA4+ pDC. A representative pDC sub-population profile out of 3 different experiments conducted separately is shown: P1-pDC (PD-L1^+^ CD80^-^) population is indicated in red, P2-pDC (PD-L1^+^ CD80^+^) in blue. The production of TNF-α (E) and IL-6 (F) was tested by cytometric bead assay in 24 hour-collected supernatants. The results shown were mean values ± SEM of 3 independent experiments. ANOVA p value for TNF-α: 0.003; for IL-6: 0.001. Based on LSD (equivalent to no adjustments).

We next sought to identify other immune cells that can sense the presence of viral RNA via TLR7/8, namely monocytes and pDC [21], and that can contribute to TLR7-driven B cell-mediated immune response via IL-6, BAFF and IFN-α production [22,23]. To this aim, we compared the levels of IFN-α released in response to I-TBEV in total PBMC and PBMC depleted of either pDC (PBMC-pDC) or monocytes (PBMC-Mo) (**Fig 3A**). IFN-α levels were poorly induced in PBMC-pDC and moderately induced in PBMC-Mo delineating pDC and monocytes as the most prominent contributors of IFN-α in our system.

The formal proof of TLR7/8 involvement in TBEV-driven effects on innate immune response was given by using a synthetic antagonist (ODN 2087), which specifically binds to and blocks TLR7/8 activity. As shown in Fig 3B, the specific inhibition of TLR7/8 strongly reduced I-TBEV-driven production of type I IFN, thus confirming the crucial role of TBEV RNA in triggering innate immune responses through recognition by TLR7/8-mediated pathway (**Fig 3B**).

The results shown in Fig 3A and 3B and the lack of type I IFN secretion in isolated monocytes following I-TBEV stimulation (**S3 Fig**) prompted us to further investigate the response of isolated pDC to I-TBEV stimulation (**Fig 3C**). As expected, I-TBEV promoted a robust production of IFN-α by pDC, which was significantly reduced in presence of ODN 2087, confirming that this cell type can sense I-TBEV via TLR7.

Interestingly, flow cytometric analysis of co-stimulatory/maturation-related (CD80, CD86, HLA-DR), and inhibitory (PD-L1, ILT7) markers as well as lineage-specific (BDCA2, BDCA4) molecules showed that I-TBEV induced a cell diversification preferentially towards a sub-population specialized in type I IFN production (called P1-pDC, 41.22% of total BDCA4^+^ pDC) (**Fig 3D**). This population was already described in other infection settings [24] and is characterized by the exclusive expression of PD-L1 (defining marker for P1-pDC) and absence of CD80 (**Fig 3D**). The presence of the TLR7/8 inhibitor reduced the percentage of P1-pDC to a level similar to unstimulated cells (19.57%), while promoting the differentiation towards P2-pDC, a subset that expresses both PD-L1 and CD80 and that has reduced ability to produce type I IFN but displays adaptive functions [24] (**Fig 3D**). This phenotypic diversification was also supported by the exclusively increased expression of the maturation markers HLA-DR and CD86, besides CD80, in I-TBEV-treated pDC exposed to TLR7/8 inhibitor. PDL1^+^ P1-pDC are instead characterized by lower levels of the inhibitory molecules BDCA2 and ILT7, known to dampen type I IFN release upon TLR-induced pDC activation [25,26] (**Figs 3D (lower panels), and S4**).

Another interesting piece of data in accordance with the stimulus-specific specialization of pDC towards IFN-producing cells in the presence of I-TBEV, emerged from the analysis of the pro-inflammatory cytokines, Tumor Necrosis Factor-Alpha TNF-α and IL-6, in pDC culture supernatants (**Fig 3E and 3F**). Both TNF-α and IL-6, factors normally released during DC maturation, are poorly induced by I-TBEV. Conversely, in the presence of TLR7/8 inhibitor, pDC displayed the adaptive P2-pDC profile with strong release of these cytokines, thus suggesting that an autocrine or paracrine IFN-α response could control the pDC pro-inflammatory cytokine profile.

### I-TBEV stimulated pDC contribute to total IgM production

**Fig 4:**
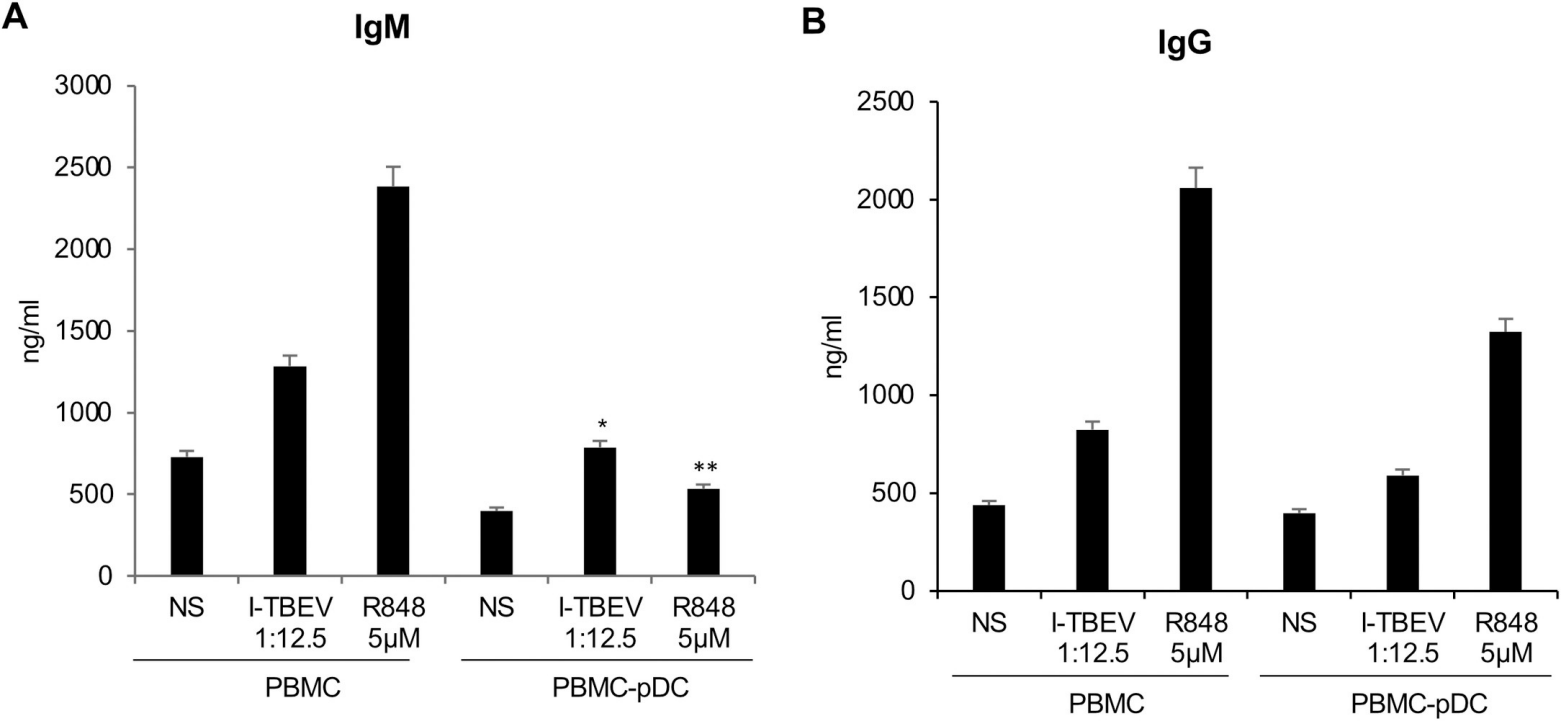
Ig production in PBMC or PBMC depleted of pDC after stimulation with I-TBEV. Total PBMC or PBMC depleted of pDC (PBMC-pDC) were left untreated (NS) or stimulated with R848 (5 μM) and inactivated TBEV (I-TBEV) (dilution 1:12.5) for 10 days. The production of either total IgM (A) or IgG (B) was measured in culture supernatants by ELISA. The results shown were mean values ± SEM of 3 independent experiments. P-value for IgM: *p = 0.03; **p = 0.02.

IL-6 and IFN-α display synergistic action on the differentiation of B cells into Ig-secreting plasma cells [27] and the type I IFN signature is associated with the production of neutralizing Ab after vaccination [28,29]. Thus, in the context of *in vitro* I-TBEV stimulation we evaluated the contribution of pDC in mediating Ig production via IFN-α release (**Fig 4**). PBMC and PBMC depleted of pDC were cultured in presence of I-TBEV and after 10 days of culture, supernatants were harvested. pDC depletion negatively affected I-TBEV capacity to promote IgM release (**Fig 4A**), while IgG production was only slightly reduced (**Fig 4B**). A similar production profile was observed in R848 treated cultures, thus supporting the hypothesis that type I IFN released by pDC contributes to TBEV-stimulated induction of IgM. Interestingly, the secretion of IgM and IgG in pDC-depleted PBMC was partially rescued by addition of IFN-α to I-TBEV-stimulated cultures (**S5 Fig**). In contrast, when IFN-α was added to Encepur-treated PBMC culture, no increase in total antibody production was observed and this is likely due to aluminum hydroxide biological interference (**S2 Fig**). Since PBMC were derived from TBEV naïve subjects, it is likely that a TBEV-specific Ab production does not occur in this setting.

### Heat-treated I-TBEV loses the capacity to stimulate IFN-α and Ig production

In an attempt to identify the viral molecules playing a role in the interaction of I-TBEV with immune cells, we initially studied in PBMC the effect of the molecular alteration generated by I-TBEV heat treatment on both antiviral response and total IgM and IgG production.

**Fig 5:**
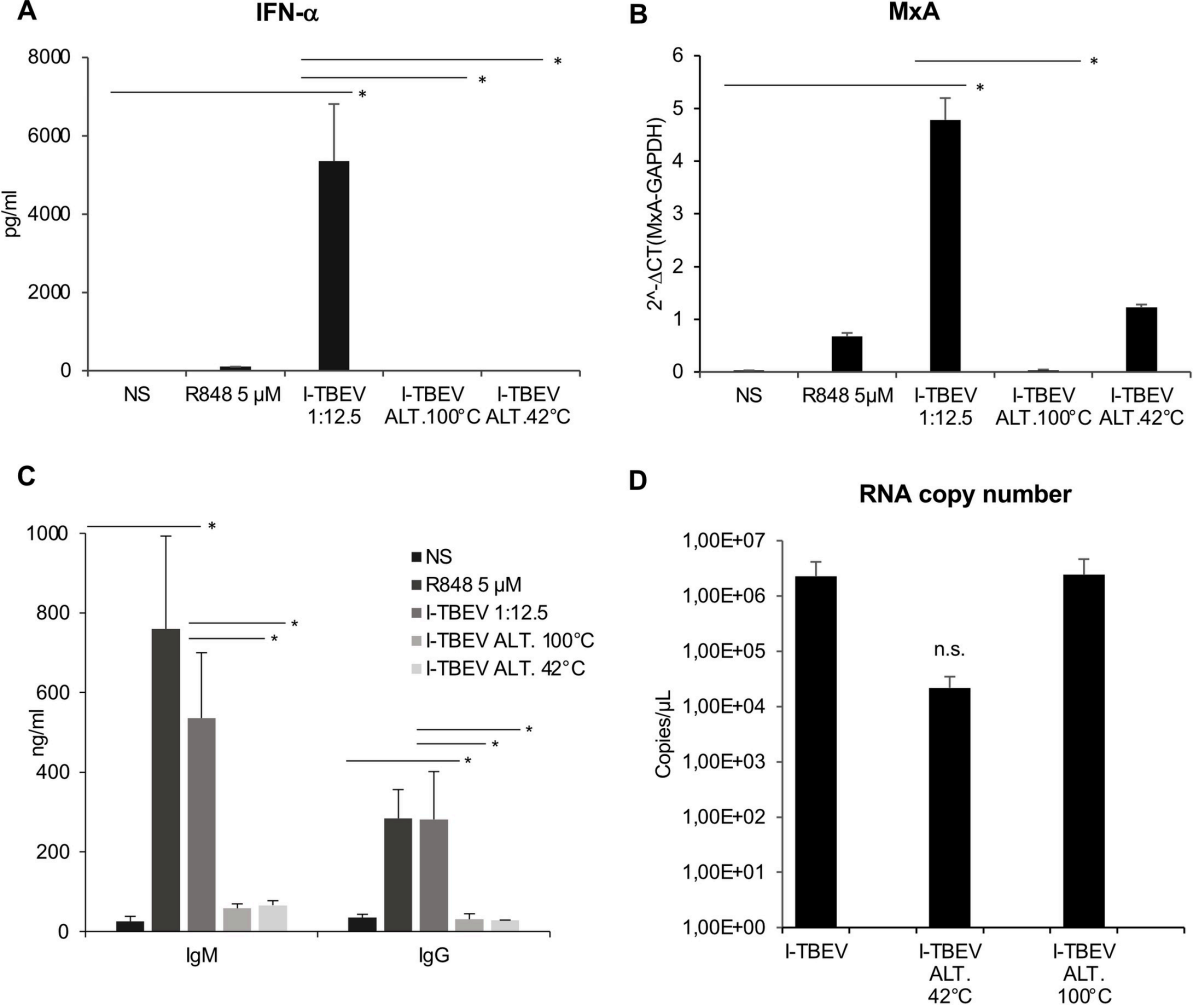
Type I IFN, MxA and Ig expression in PBMC stimulated with I-TBEV altered by temperature. PBMC were left untreated (NS) or stimulated with R848 (5 μM), I-TBEV (dilution 1:12.5) and I-TBEV altered by treatment for 10 minutes at 100°C (I-TBEV ALT 100°C) or for 4 weeks at 42°C (I-TBEV ALT 42°C). (A) The production of IFN-α was measured by ELISA in culture supernatants collected after 24 hours. The results shown were mean values ± SEM of 4 independent experiments. ANOVA p value for IFN-α: 0.012. Based on LSD (equivalent to no adjustments). (B) Relative expression of MxA on 24 hours-collected RNA samples was measured by q-PCR analysis. All quantification data are normalized to the GAPDH level by using the equation 2^−ΔCt^. The results shown were mean relative values ± SEM of 3 independent experiments. ANOVA p value for MxA: 0.000. Based on LSD (equivalent to no adjustments). (C) The levels of total IgM and IgG were measured by ELISA in culture supernatants collected after 10 days of stimulation. The results shown were mean values ± SEM of 3 independent experiments. ANOVA p value for IgM: 0.033; for IgG: 0.003. Based on LSD (equivalent to no adjustments). (D) Viral RNA copy number was measured by digital PCR analysis on I-TBEV preparations before and after temperature treatment. The results shown were mean values ± SEM of 2 independent experiments. n.s.: not significant.

To this end, I-TBEV was incubated for 10 minutes at 100°C or for 4 weeks at 42°C. Altered I-TBEV preparations were used to stimulate PBMC in comparison with the non-altered I-TBEV. By analysing type I IFN signalling as readout for innate immunity, we observed that both temperature alteration protocols significantly impaired the capacity of I-TBEV to stimulate IFN-α secretion (**Fig 5A**) and, accordingly, MxA gene expression (**Fig 5B**) as well as the release of CXCL10, being both ISGs (**S6A Fig**). Well in line with these findings and with a reduced expression of IL-6 and BAFF transcripts (**S6B and S6C Fig**), total IgM and IgG production were strongly reduced in PBMC stimulated with both preparations of heat-treated I-TBEV compared to I-TBEV (**Fig 5C**). Overall, these data indicate that PBMC can sense alterations of viral antigen induced by temperature and that antigen integrity is crucial for the stimulation of both innate and adaptive immunity.

With TBEV RNA being identified as a key molecule triggering both innate and humoral responses, we investigated whether the lack of IFN-α and Ig secretion in PBMC stimulated with heat-treated I-TBEV could be ascribed to degradation of viral RNA. Digital PCR analysis was applied on I-TBEV preparations before and after heat treatment (**Fig 5D**). Interestingly, no modification in viral RNA content among untreated or 100°C treated I-TBEV was observed, while a slight reduction occurred in 42°C altered I-TBEV.

The reduced IFN-α and Ig production elicited by heat-altered I-TBEV in spite of the presence of intact viral RNA suggests that RNA molecules are necessary but not sufficient by themselves to trigger immune stimulation thus, implying that other viral factor(s) are needed to promote pDC and B cell stimulation.

### TBEV E glycoprotein is necessary to stimulate pDC and B cell responses

**Fig 6:**
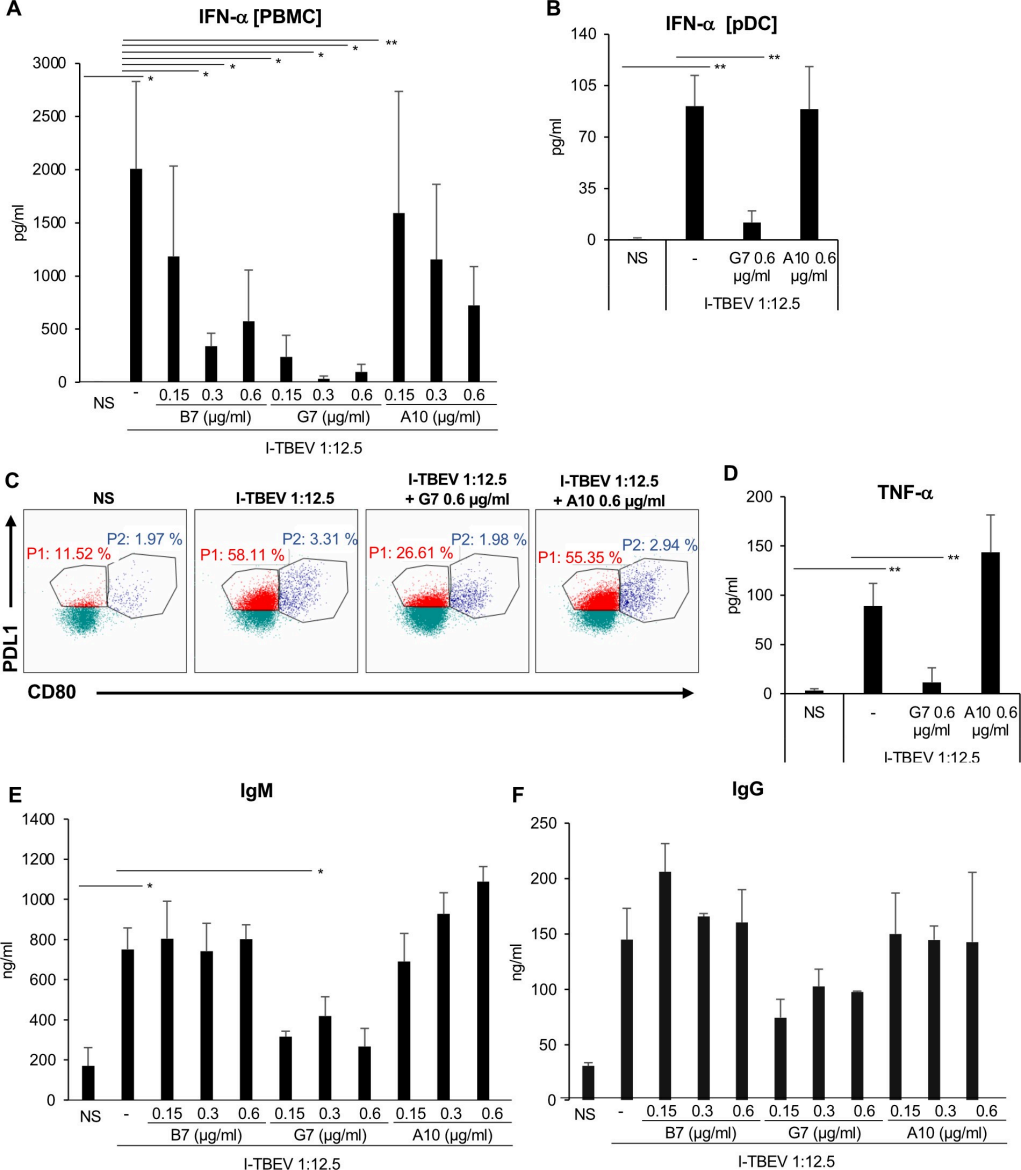
Impact of TBEV E glycoprotein neutralization on IFN-α and Ig production. Total PBMC or isolated pDC were left untreated (NS) or stimulated with I-TBEV (dilution 1:12.5) alone or in combination either with two single-chain Ab (scAb) blocking TBEV E glycoprotein, B7 and G7 or with a scAb non-related to TBEV, A10 clone. (A) The release of IFN-α was measured by ELISA in culture supernatants of total PBMC left untreated (NS) or stimulated for 24 hours with I-TBEV alone or in combination either with B7 and G7 or with a non-related scAb A10 clone at the concentration of 0.15 μg/ml, 0.3 μg/ml and 0.6 μg/ml. The results shown were mean values ± standard error of the mean (SEM) of 3 independent experiments. ANOVA p value for IFN-α: 0.006. Based on LSD (equivalent to no adjustments). (B-D) Isolated pDC were left untreated (NS) or stimulated with I-TBEV alone or in combination either with G7 clone (0.6 μg/ml) or with A10 clone (0.6 μg/ml) for 24 hours. The production of IFN-α (B) was measured by ELISA in culture supernatants. The results shown were mean values ± SEM of 3 independent experiments. ANOVA p value for IFN-α: 0.000. Based on LSD (equivalent to no adjustments). (C) pDC were stained with PD-L1, HLA-DR, CD86, CD80, ILT7, BDCA2 and BDCA4. A total of 50.000 cells were analyzed per sample by flow cytometry to evaluate the percentage of pDC sub-populations. A representative pDC sub-population profile out of 3 different experiments conducted separately is shown: P1-pDC (PD-L1^+^ CD80^-^) population is indicated in red while P2-pDC (PD-L1^+^ CD80^+^) in blue. TNF-α production (D) was tested by cytometric bead assay in 24 hour-collected supernatants. The results shown were mean values ± SEM of 3 independent experiments. ANOVA p value for TNF-α: 0.002. Based on LSD (equivalent to no adjustments). (E-F) IgM and IgG production was measured by ELISA in supernatants collected from PBMC after 10 days of stimulation. The results are mean values ± SEM of 3 independent experiments for IgM and 2 independent experiments for IgG. ANOVA p value for IgM: 0.036. Based on LSD (equivalent to no adjustments).

Given the importance of the E glycoprotein for the entry of viral particle into host cells [30], we sought to investigate how the neutralization of TBEV E glycoprotein with two specific single-chain Ab (scAb), namely G7 and B7, impacts on type I IFN and Ig release (**Fig 6**). In addition, a scAb not specific for TBEV, A10, was used to provide a reliable negative control not affecting the analyzed parameters.

Interestingly, both G7 and B7 scAbs drastically reduced in a dose-dependent manner the production of IFN-α, with G7 displaying a stronger activity as compared to B7 (**Fig 6A**). A similar effect was observed in isolated pDC where the neutralization of TBEV E glycoprotein by G7 almost abolished IFN-α release (**Fig 6B**). This inhibition acts also at a phenotypical level since the diversification of pDC into PDL1^+^ IFN-producing P1 subset was completely reverted when E glycoprotein was neutralized (**Figs 6C and S7**). Of note, TNF-α production was also drastically reduced when TBEV E glycoprotein was neutralized by G7 addition (**Fig 6D**).

Blocking of TBEV E glycoprotein by G7 also affected Ig production induced in PBMC by I-TBEV stimulation (**Fig 6E and 6F**), with a significant reduction particularly in IgM release (**Fig 6E**). No changes were observed when the negative control A10 scAb was used.

Collectively, these data indicate that cooperation between E glycoprotein and viral RNA acting in tandem is crucial for the activation of both anti-viral and humoral immune responses induced by TBEV: the E glycoprotein is necessary for binding of the virus to the cells allowing/facilitating virus entry of virus and delivery of viral RNA molecules to endosomal compartment for a proper TLR7 stimulation.

## Discussion

Although vaccination against TBEV is generally considered effective and has decreased the incidence of the disease in endemic regions, TBE cases among vaccinated subjects have been reported [31–33], thus a better understanding of the immune response to the vaccine is critical for identifying reliable correlate/s of vaccine efficacy and key viral immuno-stimulatory molecules. Therefore, we investigated the impact of Encepur, one of the five TBEV vaccines currently available, and its single components on the stimulation of human PBMC, which harbor the majority of immune cells responding to vaccination. Indeed, the results of this study provide interesting cues on the interlink between B cell response and the cytokines released by pDC that are likely to get activated and recruited *in vivo* at the injection site by aluminum-adjuvanted TBEV vaccine. This hypothesis relies on data obtained in mice injected intraperitoneally with ovalbumin emulsified in alum adjuvant where pDC, together with other innate immune cells, were recruited into the peritoneal cavity 12 and 24 h after injection [34,35]. Unfortunately, in this PBMC-based experimental setting we were not able to study Encepur, the final vaccine formulation. This limitation is likely due to the presence of aluminum hydroxide in Encepur that alters the *in vitro* cellular response, and this may provide an incomplete picture of what occurs *in vivo* after vaccination. Future investigation in *in vivo* potency test performed in mice challenged with live TBEV will help in providing an *in vivo* proof of evidence of our *in vitro* study. However, in the human PBMC-based *in vitro* experimental setting we were able to collect important information on the viral components involved in immune stimulation by mimicking the scenario that on one hand TBEV may face during the viremic phase of its replication, and on the other hand TBEV vaccine may encounter following the interaction with specific leukocyte populations that are involved in antiviral immunity.

TBEV E glycoprotein and RNA were found to be crucial and to cooperate for the stimulation of target immune cells. Interestingly, previous reports showed that a similar dual stimulation of pDC leading to type I IFN production, occurs also with protein A, a surface protein with *S*. *aureus*, and bacterial DNA [36] as well as in response to inactivated whole-influenza virus vaccine containing both viral protein and RNA [37]. Based on our data we propose the following model: E glycoprotein is required for both receptor binding and fusion to host cell thus allowing viral RNA to reach the endosomal compartment for TLR7 stimulation, together with other immunogenic components. Given the high degree of amino acid similarity among the TBEV strains in the E protein (77–98%), especially in domain III (80–95%) [38,39] and TLR7 being a pattern recognition receptor that broadly recognizes single strand RNA structures, what we observed with the Karlsruhe (K23) strain, used in Encepur, likely occurs also with the TBEV strains included in the other vaccines.

As a main target for host immune humoral responses, the importance of E proteins of TBEV and other flaviviruses has been well characterized in the last years [40], while the role of viral RNA in promoting anti-TBEV immune response has not been studied so far. Since TBEV is a single-stranded RNA virus, we hypothesized that I-TBEV, being killed by formaldehyde and thus unable to form double stranded (ds)RNA structures targeting TLR3, stimulates mainly TLR7-driven innate immune responses. In line with this hypothesis, in murine neurons TLR7 triggering was shown to suppress replication of Langat virus (LGTV), a naturally attenuated member of the TBEV serogroup [41]. Accordingly, the importance of TLR7 in response to the infection with single-stranded RNA viruses was repeatedly demonstrated in mouse models. Different studies demonstrated that the lack of this receptor in TLR7-deficient mice or the block of the TLR7-signaling cascade has a detrimental impact on the activation of pDC and B cells as well as on the production of cytokines, including type I IFN [42–44]. Indeed, among human TLR7-expressing cells present in human PBMC, pDC and B cells are particularly responsive to TLR7 agonists, which induce important phenotypic and functional changes in these leukocyte populations [45,46]. Type I IFN-stimulated antiviral response and IgM and IgG production were investigated showing that both cell types were involved in the establishment of I-TBEV-stimulated immune responses.

The analysis of B cell response in PBMC of TBEV vaccine-naïve healthy donors to the stimulation with Encepur and its components highlighted that I-TBEV significantly promoted B cell differentiation in Ab-producing plasmablasts and, in turn, total IgM and IgG production, while Encepur failed to do so in this *in vitro* setting. Nevertheless, anti-TBEV specific Ab could not be detected in our setting, since virus-specific memory B cells are unlikely to be present in the blood donors enrolled in this study, likely not infected with TBEV and not vaccinated. Memory B cells were previously shown to respond more readily to TLR stimulation once the BCR has been triggered and the nucleic acid has received access to the endosome [22,47–49].

However, B cell differentiation was dependent on type I IFN induction and TLR7-mediated recognition of viral RNA, which resulted in release of IgM. This was not surprising because type I IFN was previously shown to induce TLR7 expression in B cells, thereby sensitizing them for TLR7 ligands, which induced naïve B cell differentiation into unspecific IgM-secreting cells [22]. In the absence of TLR7 stimulation and type I IFN, as in the case of Encepur stimulation, no IgM secretion was detectable.

Since the treatment with excipient alone was also ineffective in modulating both total Ig production and plasmablast differentiation, we assume that in our *in vitro* setting aluminum hydroxide, in spite of being used as potent enhancer of anti-TBEV neutralizing Ab production *in vivo* [50], alters the B cell immunostimulatory effects of Encepur as compared to I-TBEV. Indeed, differently from *in vivo* administration where most antigens can be released from the surface of aluminum hydroxide-based adjuvants and rapidly leave the injection site, the presence of aluminum hydroxide in cell culture condition could mask important epitopes or structures of the antigen necessary for immune cell stimulation. Moreover, the lack of type I IFN in PBMC and the high mortality rate of isolated pDC following Encepur treatment, might explain the poor Ig production and plasmablast differentiation. Alternatively, aluminum hydroxide could exert inhibitory impact directly on B cell response or *via* a bystander effect on BAFF or IL-6 producing cells, as monocytes [51].

Conversely, I-TBEV was a strong inducer of CD19^+^CD27^++^CD38^++^ plasmablast differentiation via the induction of the transcription factor BLIMP1, and its target gene XBP1, and the induction of a favourable cytokine milieu, containing BAFF and IL-6 as well as type I IFN. Interestingly, in addition to the well-characterized importance of IL-6 and BAFF in sustaining B cell-mediated immunity [27,52–54], only recently type I IFN has been demonstrated by a systems biology approach to be predictive of an immune response in humans vaccinated with yellow fever vaccine YF-17D [55] or with seasonal influenza vaccine [56]. Transcriptional profiling of total PBMC from the vaccinated subjects showed the modulation of molecules involved in innate sensing of viruses as well as transcription factors that regulate type I IFN thus indicating that processes related to innate immunity may have influenced the immunogenicity of either vaccine. By depleting pDC, the major cells producing type I IFN in response to I-TBEV, a reduction of total Ig production was observed likely conditioned also by the reduced type I IFN levels in accordance with literature data showing the importance of synergistic action of pDC-released IL-6 and IFN-α/β for Ig-producing plasma cell differentiation [27]. Moreover, as demonstrated for two other Flaviviruses—Dengue and Zika viruses—whose E glycoprotein undergoes conformational alteration when subjected to moderate temperature treatments [57–59], it is likely that this occurs also for the head-to-tail dimer structure of the TBEV native E glycoprotein [60]. Temperature-dependent changes of TBEV E glycoprotein, in turn, might be responsible for an impaired type I IFN, IL-6 and BAFF expression and reduced B cell stimulation. So, these data are also reminiscent of previous results showing that in the presence of RNA containing immune-complexes the pDC-B cell crosstalk leads to B cell expansion [61], and support the assumption that release of type I IFN by pDC could be predictive of immunogenicity of vaccines, as influenza virus vaccine [37] and, in our case, of I-TBEV [62]. Interestingly, these results on the possibility to monitor type I IFN may have also some relevance for setting up a cell-based platform to evaluate *in vitro* the potency of TBEV vaccine batches, prior to the aluminum hydroxide adsorption, in order to identify low-quality non-compliant batches and, thus, reducing animal testing so far mandatory for vaccine batch release. Moreover, these data could also provide important clues for the assessment of critical parameters, namely type I IFN release, that are still not well-characterized as putative host-target of viral vaccine.

In addition, we found that TBEV induces also IFNL1 in human PBMC mirroring which was observed in human medulloblastoma-derived neuronal cell line (DAOY) [9]. However, we do not know whether in our experimental model IFNL1 synergizes with type I IFN in controlling virus replication or if it acts to dampen the pro-inflammatory response [63].

Our study, hence, shows that native TBEV structure and, in particular, intact viral RNA and E glycoprotein are not only important for B cell response but also critical for pDC activation. In particular, the majority of I-TBEV-treated pDC display a phenotypical diversification towards PD-L1^high^ P1-pDC specialized in type I IFN production, reminiscent of innate-like specialization [24]. When in I-TBEV-treated cultures TLR7/8 signaling was inhibited by a specific oligodeoxynucleotide antagonist, pDC changed their phenotype towards a PD-L1^+^CD80^+^ P2-pDC more specialized in adaptive function and T cell commitment and inflammatory cytokine production. Indeed, pDC in presence of TLR7/8 inhibitor express high level of TNF-α which is known to cross-regulate with IFN-α production [64]. When TBEV E glycoprotein was blocked by G7, a specific blocking scAb, the high expression of PD-L1 observed in I-TBEV-treated pDC completely reverted, however their phenotype did not turn into adaptive CD80^+^ P2-pDC indicating that in the absence of E glycoprotein signalling I-TBEV did not stimulate or activate at all pDC, as instead was observed when TLR7/8 pathway was blocked. Although it is not clear so far whether the addition of blocking scAb impacts not only on the binding of E glycoprotein with the not-yet identified cellular receptor, but also on virus uptake and compartmentalization—as demonstrated in Dengue infection of murine macrophages [65]—our data are suggestive of the importance of the E glycoprotein in the host cell entry and, subsequently, in the fusion of viral and cellular membranes within the acidic endosome environment. It is, thus, likely that the viral RNA molecules, via TLR7 stimulation, are mainly responsible for PD-L1^high^ P1-pDC innate functions, while other viral structures may support the PD-L1^+^CD80^+^ P2-pDC maturation process and adaptive function.

To our knowledge, here we describe for the first time the ability of TBEV to stimulate pDC via TLR7 and the role of these cells in initiating both antiviral and B cell-mediated immune responses via type I IFN production in the context of TBEV infection. The combined action of the E glycoprotein and the viral RNA is crucial for a successful pDC activation and type I IFN release, that together with TLR7 stimulation promote B cell response. In order to properly stimulate naïve B cell differentiation during the primary vaccination, the TLR7/type I IFN axis needs to be preserved for optimal immune response induction and taken into consideration in case of a novel manufacturing process of inactivated vaccines or simpler vaccine formulations than the current whole inactivated TBEV-based vaccine.

## Materials and methods

### Ethic statement

Istituto Superiore di Sanità Review Board approved the use of PBMC from healthy volunteers (CE/13/387) for this study. No informed consent was given since anonymous blood bags were kindly donated by the Blood Transfusion Service and Hematology department of Umberto I Hospital (Rome, Italy).

### Reagents

The Toll-like receptor (TLR)7/8 antagonist, ODN 2087, was purchased from Miltenyi Biotech, (Bergisch Gladbach, Germany). Single-chain Fragment variable–Fragment crystallizable antibodies (scAb) specific for TBEV E glycoprotein, B7 and G7 clones, or non-specific as A10 clone were generated by Yumab (Braunschweig, Germany) and provided by GSK.

### Vaccine samples

Encepur vaccine now is a trademark of Bavarian Nordic while at the beginning of this study it was a GSK trademark. Encepur is manufactured using the TBEV-Eu strain K23, that is inactivated with formaldehyde and then purified on sucrose gradient. The active drug substance of Encepur is the inactivated TBEV, herein named I-TBEV, whose content in a vaccine dose is 1.5 μg (3 μg/ml) [66,67].

The excipient matrix (herein named excipient), containing aluminum hydroxide, possesses physiochemical properties and chemical composition similar to the drug product but does not contain the I-TBEV, thus was used as vaccine control. A 2.1% low-endotoxin sucrose (Sigma-Aldrich, St. Louis, USA) solution was prepared in RPMI 1640 medium (BioWhittaker Europe, Verviers, Belgium) and used as control for I-TBEV (herein named sucrose). Where indicated, I-TBEV was altered by heat treatment at 42°C for 4 weeks or at 100°C for 10 minutes. To perform a reliable comparison between the vaccine (final formulation) and its single components, all the analyzed stimuli were used at the same final concentration to treat PBMC. The volume/volume ratio was used to indicate treatment conditions with the same amount of stimuli.

### Isolation and stimulation of PBMC and pDC

PBMC were collected from healthy donors and isolated and cultured at 2x10^6^/ml as described [68]. pDC were purified from isolated PBMC as previously described [69]. The purity of the recovered cells was greater than 95% as assessed by flow cytometry analysis with an anti-BDCA4 monoclonal Ab (Miltenyi biotech). Isolated pDC were plated at a density of 1x10^6^/ml and, for each experimental condition, 1x10^5^ pDC were used.

For pDC or monocyte cell depletion, PBMC were subjected to positive sorting using anti-BDCA-4 (CD304) or anti-CD14 conjugated magnetic microbeads (Miltenyi Biotech), respectively. The eluates, containing depleted populations, were collected.

Whole PBMC were stimulated with the 1:12.5 and 1:50 dilutions of Encepur and I-TBEV corresponding to 0.24 μg/ml and 0.06 μg/ml of antigen, respectively. Same dilutions were used for the excipient and sucrose solution. The TLR7/8 ligand Resiquimod (R848, 5 μM, Invivogen San Diego, CA), was used as positive control. Cells were stimulated for the indicated time, supernatants collected, and RNA extracted for further analyses. Where indicated, cells were also pre-treated for 30 minutes at 37°C with 1 μM TLR 7/8 antagonist ODN 2087 (Miltenyi Biotech) prior to I-TBEV stimulation. Where specified, I-TBEV was incubated with scAb (0.15, 0.3 or 0.6 μg/ml) against TBEV for 30 minutes at 37°C before PBMC treatment.

### Flow cytometric analysis

Monoclonal Ab specific for CD14, CD19, CD38, CD27, PD-L1, CD80, CD86, IL-T7, HLA-DR, CD123 as well as IgG1 or IgG2a isotype controls were purchased from BD Biosciences (San Diego, CA, USA), BDCA2 from Biolegend (Fell, DE), BDCA4 from Miltenyi Biotech and IgM by Jackson Laboratories (Bar Harbour, Maine, USA). To establish viability of cells and to exclude dead cells from flow cytometry analysis Fixable Viability Dye eFluor780 (FvDye) (eBioscience, San Diego, CA, USA) was used as previously described [68]. Briefly, cells (10^5^ for PBMC and 5x10^4^ for isolated pDC) were incubated with monoclonal Abs at 4°C for 30 min and then fixed with 2% paraformaldehyde before analysis on a Gallios cytometer (Beckman Coulter). Data were analyzed by Kaluza software (Beckman Coulter). The expression of cell surface molecules was evaluated using the median fluorescence intensity (MFI) after subtraction of the values of the isotype Ab controls. Only cells present in the viable cell gate were considered for the analysis.

### Detection of cytokines, chemokines and immunoglobulins

Supernatants from cell cultures were harvested after stimulation as described and stored at -80°C. The release of IFN-α (PBL assay science, NJ, USA), total IgM and IgG, (Bethyl Laboratories, Inc., Montgomery, TX, USA) and CXCL10 (R&D Systems, Minneapolis, MN, USA) was measured by specific ELISA kits, while the production of IL-8, IL-6 and TNF-α was quantified by Cytometric Bead Assay (BD Biosciences, San Diego, CA, USA).

### RNA purification and real-time RT-PCR

RNA was isolated from PBMC by using TRIzol Reagent (Invitrogen, Life Technologies) according to the manufacturer’s instructions. Reverse transcription and quantitative PCR assay were performed as previously described [70]. Primers used for IFNA (corresponding to the common sequence of the 12 IFN-α subtypes), IFNB1, Cytomegalovirus Induced Gene 5 (CIG5), Myxovirus Resistance Protein-1 (MxA), B-Cell-Activating Factor (BAFF), TATA-Box Binding Protein (TBP) and Glyceraldehyde-3-Phosphate Dehydrogenase (GAPDH) were previously described [51,70–72], while those for B-Lymphocyte-Induced Maturation Protein 1 (BLIMP1) and X-Box-Binding Protein 1 (XBP1) were as follows:

BLIMP1 FOR: TGCGGATATGACTCTGTGGA

BLIMP1 REV: ACGTGTGCCCTTTGGTATGT

XBP1 FOR: TCTGGCGGTATTGACTCTTC

XBP1 REV: GAGAAAGGGAGGCTGGTAAG

The expression of IFNL1, 2’-5’-oligoadenylate synthetase 1 (OAS1) and IFN Regulatory Factor (IRF) 7 was analyzed by specific TaqMan assay (Thermofisher Scientific) and TaqMan Universal Master Mix II (Thermofisher Scientific) as previously described [73]. Transcript expression was normalized to the GAPDH or TBP level quantified by threshold cycle (Ct) by using the Equation 2^−ΔCt^; the values are means ± SD of triplicate determinations.

### Droplet digital polymerase chain reaction (ddPCR)

Viral RNA was isolated from I-TBEV by QIAamp Viral RNA Mini Kit (Qiagen) following manufacturer’s instructions (except for the incubation period with the lysis buffer, which was extended to 1 h). The Primescript RT Reagent kit (Takara, Saint-Germain-en-Laye, France) was used for the reverse transcription of the isolated RNA. The ddPCR was performed following the instructions of the manufacturer using ddPCR Supermix for Probes (no dUTP), a Droplet Generator, PCR Plate Sealer, Thermal Cycler with 96-Deep Well Reaction module and QX200 Droplet Digital PCR System (all Bio-Rad, Hercules, CA). TBEV-specific primers and probe used to amplify the viral RNA were previously described [74].

### Statistical analysis

Statistical analysis was performed using One-way Repeated-Measures ANOVA when three or more stimulation conditions are compared. In case of significant ANOVA, the pairwise comparisons were carried out by the use of post-hoc approaches for multiple comparisons, in order to test the significance of the difference between two stimulation effects. A two-tailed paired Student’s t-test was used when only two stimulation conditions are compared. In all the cases above, a *p* value < 0.05 was considered statistically significant: “*” refers to the p-value ≥ 0.01 and < 0.05; “**” refers to the p-value is ≥ 0.001 and < 0.01; “***” the p-value is <0.001. Data analysis was processed by the software IBM SPSS 26.0.

## Supporting information

S1 Fig Determination of MxA copy number. MxA copy number in PBMC left untreated (NS) or stimulated with I-TBEV or Encepur vaccine (dilution 1:12.5) for 24 hours was determined by digital-PCR analysis. The results shown were mean values ± SEM of 3 independent experiments.
(TIFF)

S2 Fig Effect of aluminum and type I IFN/IL-6 on Ig secretion. PBMC were left untreated (NS) or stimulated with I-TBEV (dilution 1:12.5), alone or in combination with excipient matrix or Encepur (both at dilution 1:12.5) and Encepur (dilution 1:12.5), alone or in presence of IFNα (1000U/ml) and/or IL-6 (20 ng/ml). The levels of total IgM and IgG were measured by ELISA in culture supernatants collected after 10 days of stimulation. The results shown were mean values ± SEM of 2 independent experiments.
(TIFF)

S3 Fig Production of type I IFN in PBMC and monocytes stimulated with inactivated TBEV and Encepur. Total PBMC and monocytes isolated from the same PBMC donor were left untreated or stimulated with I-TBEV (1:12.5) or Encepur (1:12.5) for 24 hours. The production of IFN-α was measured in culture supernatants by ELISA. The results shown were mean values ± SEM of 3 independent experiments.
(TIFF)

S4 Fig Phenotypic diversification of human pDC induced by I-TBEV stimulation. Isolated pDC were left untreated (NS) or stimulated for 24 hours with I-TBEV (dilution 1:12.5) alone or in combination with a TLR-7/8 inhibitor. Two pDC sub-populations were characterized on the basis of PD-L1 and CD80 expression: P1-pDC (PD-L1^+^CD80^-^); P2-pDC (PD-L1^+^CD80^+^). The mean fluorescence intensity (MFI) of PD-L1, CD80, CD86, BDCA2, ILT7, HLA-DR and BDCA4 molecules was determined by flow cytometer analysis. The results shown are mean values ± standard error of the mean of 2 independent experiments.
(TIFF)

S5 Fig Effect of type I IFN on Ig production in pDC-depleted PBMC after I-TBEV stimulation. PBMC depleted of pDC (PBMC-pDC) were left untreated (NS) or stimulated with inactivated TBEV (I-TBEV) (dilution 1:12.5) alone or in combination with IFN-α (1000U/ml) for 10 days. As control, total PBMC from same donor were left unstimulated or treated with I-TBEV. The production of either total IgM (A) or IgG (B) was measured in culture supernatants by ELISA. The results shown were mean values ± SEM of 2 independent experiments.
(TIFF)

S6 Fig Effect of I-TBEV temperature alteration on CXCL-10 and IL-6 production and BAFF expression. PBMC were left untreated (NS) or stimulated with R848 (5 μM), I-TBEV (dilution 1:12.5) and I-TBEV altered by temperature for 10 minutes at 100°C (I-TBEV ALT 100°C) or for 4 weeks at 42°C (I-TBEV ALT 42°C). (A-B) The production of CXCL10 and IL-6 was measured in culture supernatants collected after 24 hours by ELISA and cytometric bead assay, respectively. The results shown were mean values ± standard error of the mean (SEM) of 3 independent experiments. (C) Relative expression of BAFF was measured by q-PCR analysis on RNA extracted after 24 hours of stimulation. Data are normalized to TBP level by using the equation 2^−ΔCt^. The results shown were mean relative values ± SEM of 3 independent experiments.
(TIFF)

S7 Fig Impact of TBEV E glycoprotein neutralization on phenotypic diversification of pDC. Isolated pDC were left untreated (NS) or stimulated for 24 hours with I-TBEV (dilution 1:12.5) alone or in combination either with single-chain antibody (scAb) blocking TBEV E glycoprotein, G7 (0.6 μg/ml) or with a non-related scAb, A10 clone (0.6 μg/ml). Two pDC sub-populations were characterized on the basis of PD-L1 and CD80 expression: P1-pDC (PD-L1^+^CD80^–^); P2-pDC (PD-L1^+^CD80^+^). The mean fluorescence intensity (MFI) of PD-L1, CD80, CD86, BDCA2, ILT7, HLA-DR and BDCA4 was determined by flow cytometer analysis. The results shown are mean values ± standard error of the mean of 2 independent experiments.
(TIFF)

S1 Table Evaluation of cell mortality in human PBMC, isolated monocytes and pDC after treatment with ENCEPUR vaccine, inactivated TBEV, excipient matrix or sucrose. Peripheral blood mononuclear cells (PBMC) were left untreated (NS) or stimulated with inactivated TBEV (I-TBEV), Encepur vaccine, excipient and sucrose (1:12.5 and 1:50) for 24 hours. Isolated monocytes or plasmacytoid dendritic cells (pDC) were left untreated (NS) or stimulated with inactivated TBEV (I-TBEV), Encepur vaccine, excipient and sucrose (1:12.5) for 24 hours. Cell viability was assessed by staining PBMC with the Fixable viability Dye (FvDye). The results shown are mean values ± standard error of the mean of 3 independent experiments for PBMC and mean values ± standard error of the mean of 2 independent experiments for monocytes and pDC.
(TIFF)

S2 Table Effect of aluminum hydroxide interference on IFN-α produced by I-TBEV stimulated PBMC. (A) Technical interference of aluminum hydroxide was evaluated by adding Excipient (1:12.5) to IFN-α standard protein and then measuring the optical density value by ELISA. (B) Peripheral blood mononuclear cells (PBMC) were left untreated (NS) or stimulated with Encepur vaccine, excipient and inactivated TBEV (I-TBEV) alone or in combination either with Encepur vaccine or with excipient (1:12.5) for 24 hours. The production of IFN-α was measured in culture supernatants by ELISA. The results shown are mean values ± standard error of the mean of 3 independent experiments.
(TIFF)
