# Supplementary figures and images for: Human plasmacytoid dendritic cells at the crossroad of type I interferon-regulated B cell differentiation and antiviral response to tick-borne encephalitis virus
Source: PLoS Pathog. 2021 Apr 15;17(4):e1009505. doi: 10.1371/journal.ppat.1009505 (PMC8078780; doi:10.1371/journal.ppat.1009505)

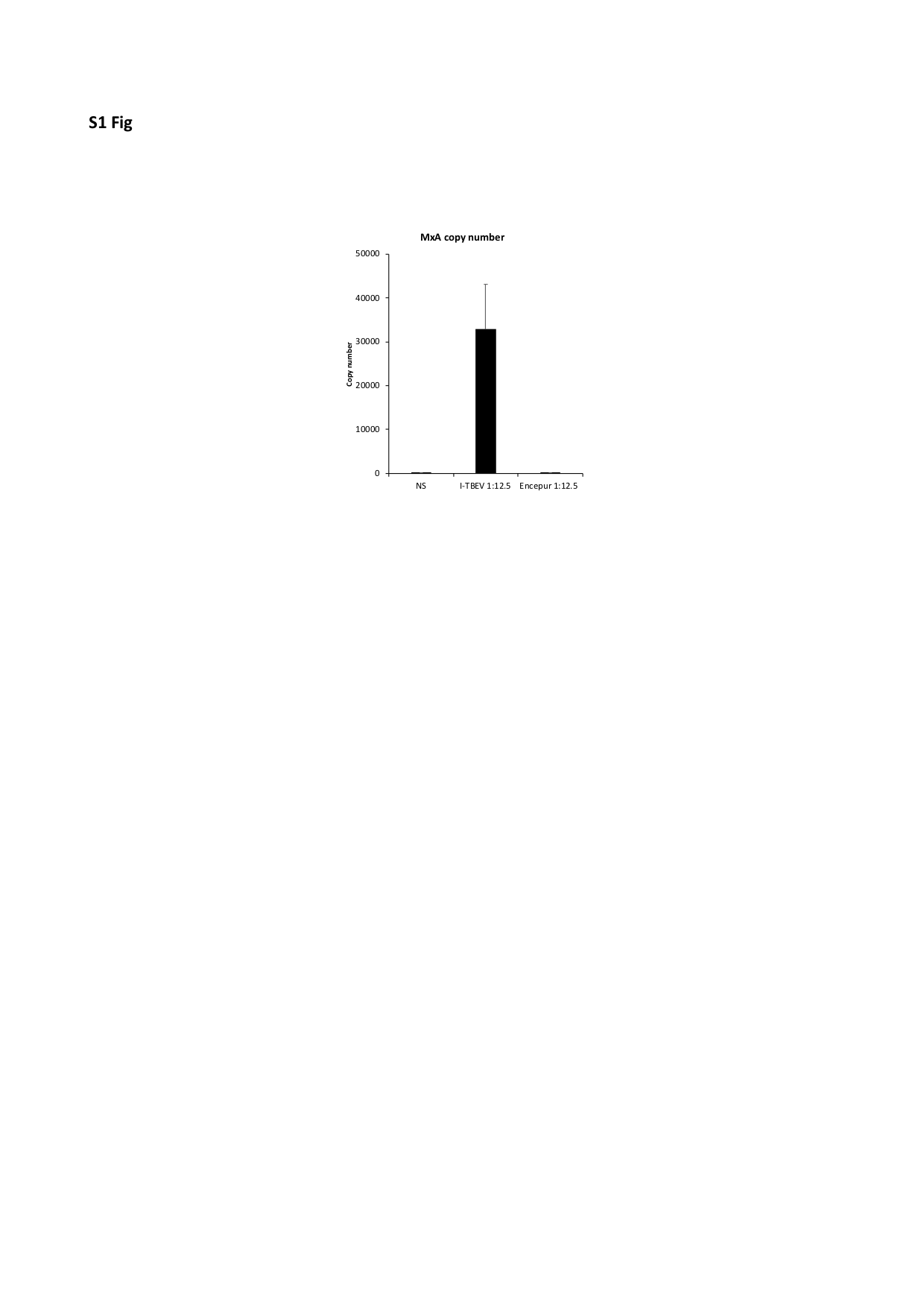

Supplement: S1 Fig — MxA copy number in PBMC left untreated (NS) or stimulated with I-TBEV or Encepur vaccine (dilution 1:12.5) for 24 hours was determined by digital-PCR analysis. The results shown were mean values ± SEM of 3 independent experiments. (TIFF) [file ppat.1009505.s001.tiff]

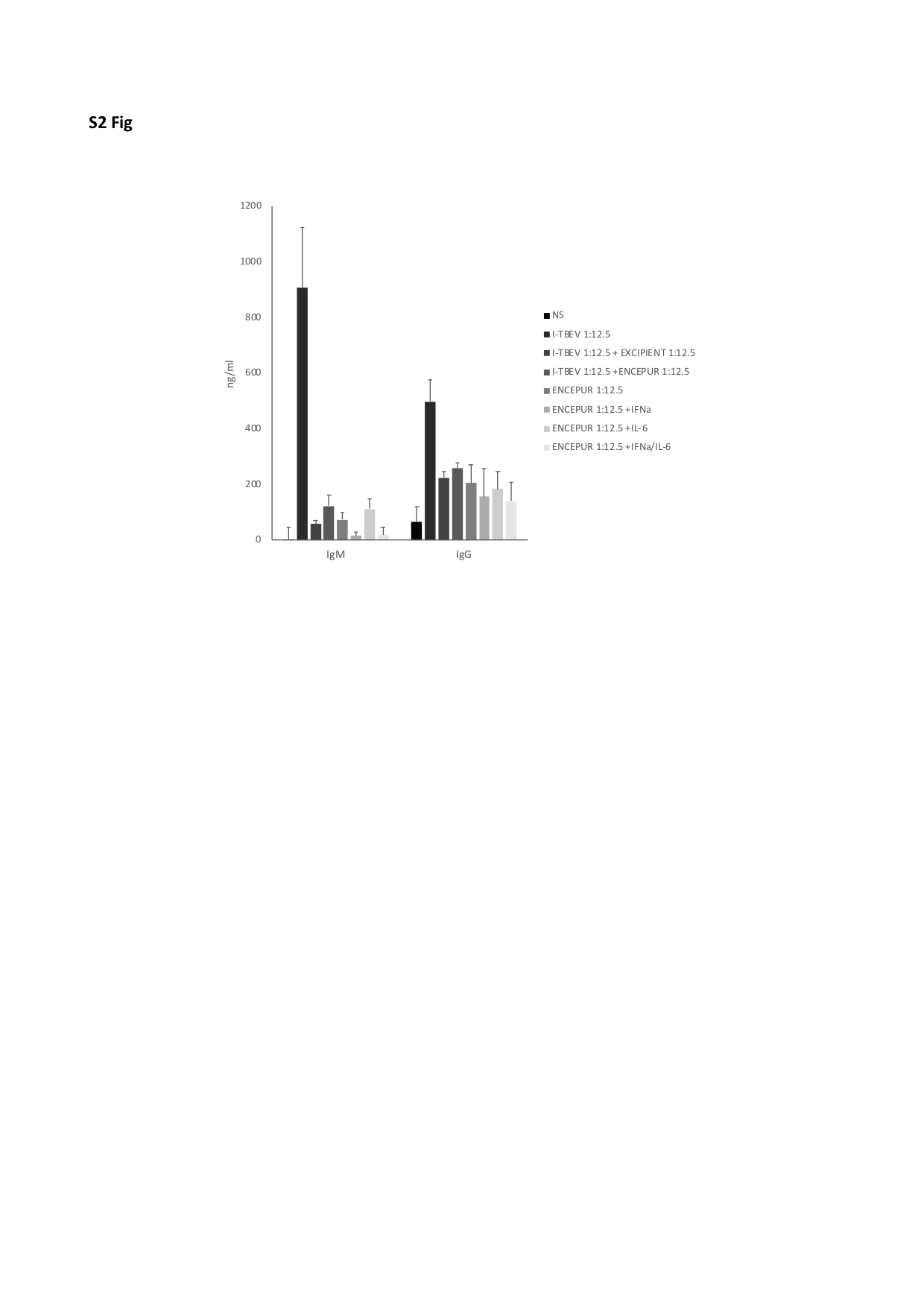

Supplement: S2 Fig — PBMC were left untreated (NS) or stimulated with I-TBEV (dilution 1:12.5), alone or in combination with excipient matrix or Encepur (both at dilution 1:12.5) and Encepur (dilution 1:12.5), alone or in presence of IFNα (1000U/ml) and/or IL-6 (20 ng/ml). The levels of total IgM and IgG were measured by ELISA in culture supernatants collected after 10 days of stimulation. The results shown were mean values ± SEM of 2 independent experiments. (TIFF) [file ppat.1009505.s002.tiff]

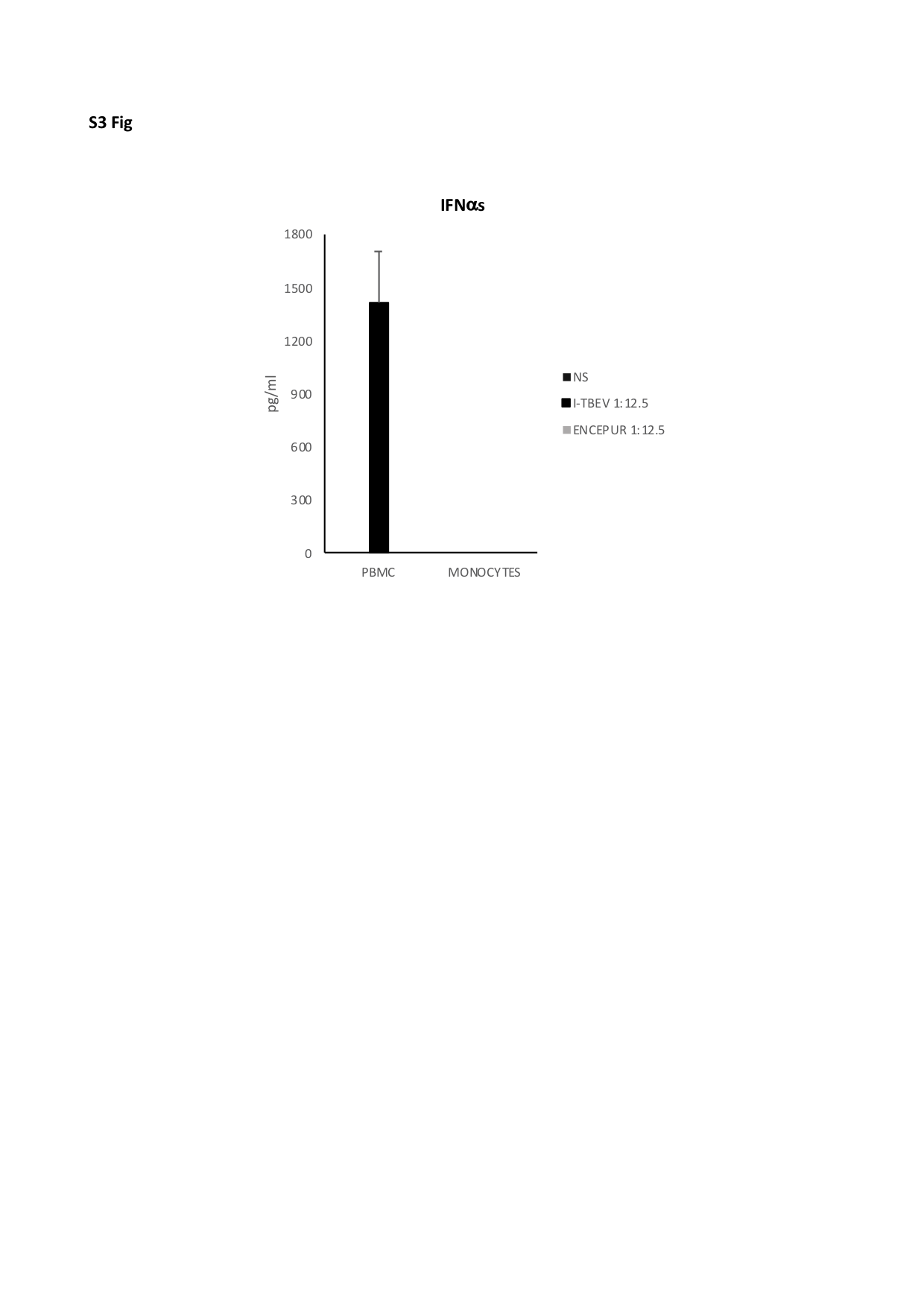

Supplement: S3 Fig — Total PBMC and monocytes isolated from the same PBMC donor were left untreated or stimulated with I-TBEV (1:12.5) or Encepur (1:12.5) for 24 hours. The production of IFN-α was measured in culture supernatants by ELISA. The results shown were mean values ± SEM of 3 independent experiments. (TIFF) [file ppat.1009505.s003.tiff]

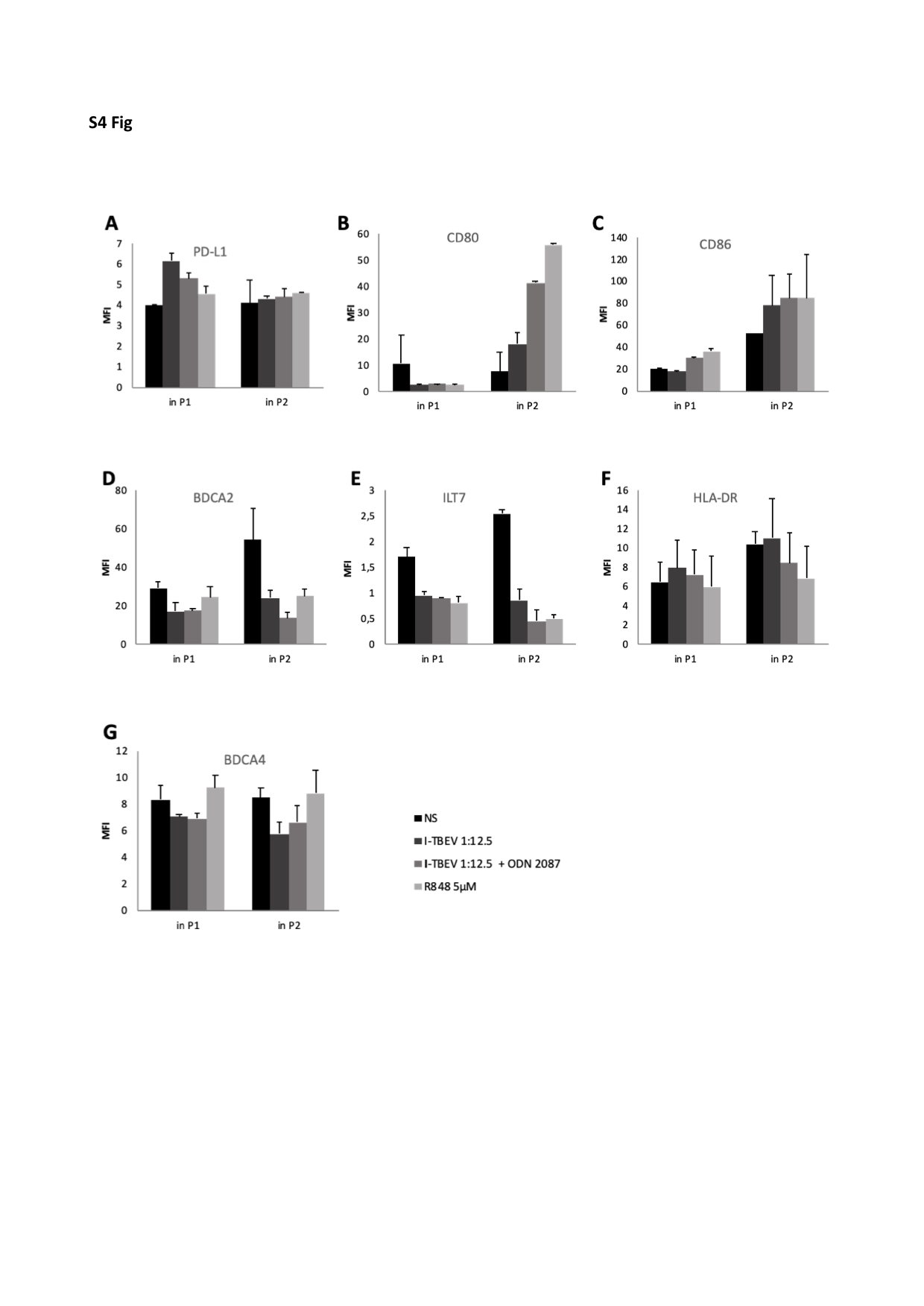

Supplement: S4 Fig — Isolated pDC were left untreated (NS) or stimulated for 24 hours with I-TBEV (dilution 1:12.5) alone or in combination with a TLR-7/8 inhibitor. Two pDC sub-populations were characterized on the basis of PD-L1 and CD80 expression: P1-pDC (PD-L1+CD80-); P2-pDC (PD-L1+CD80+). The mean fluorescence intensity (MFI) of PD-L1, CD80, CD86, BDCA2, ILT7, HLA-DR and BDCA4 molecules was determined by flow cytometer analysis. The results shown are mean values ± standard error of the mean of 2 independent experiments. (TIFF) [file ppat.1009505.s004.tiff]

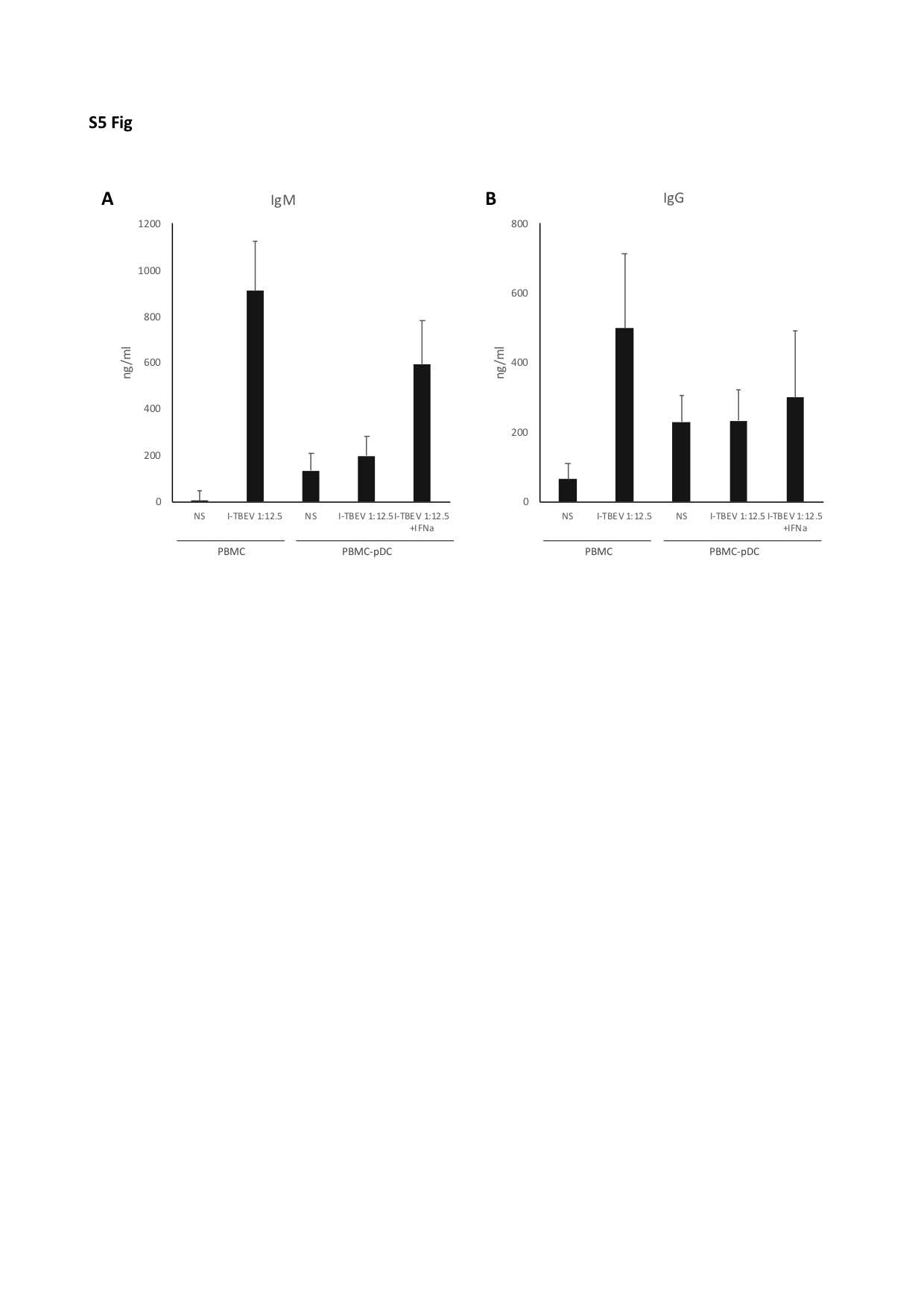

Supplement: S5 Fig — PBMC depleted of pDC (PBMC-pDC) were left untreated (NS) or stimulated with inactivated TBEV (I-TBEV) (dilution 1:12.5) alone or in combination with IFN-α (1000U/ml) for 10 days. As control, total PBMC from same donor were left unstimulated or treated with I-TBEV. The production of either total IgM (A) or IgG (B) was measured in culture supernatants by ELISA. The results shown were mean values ± SEM of 2 independent experiments. (TIFF) [file ppat.1009505.s005.tiff]

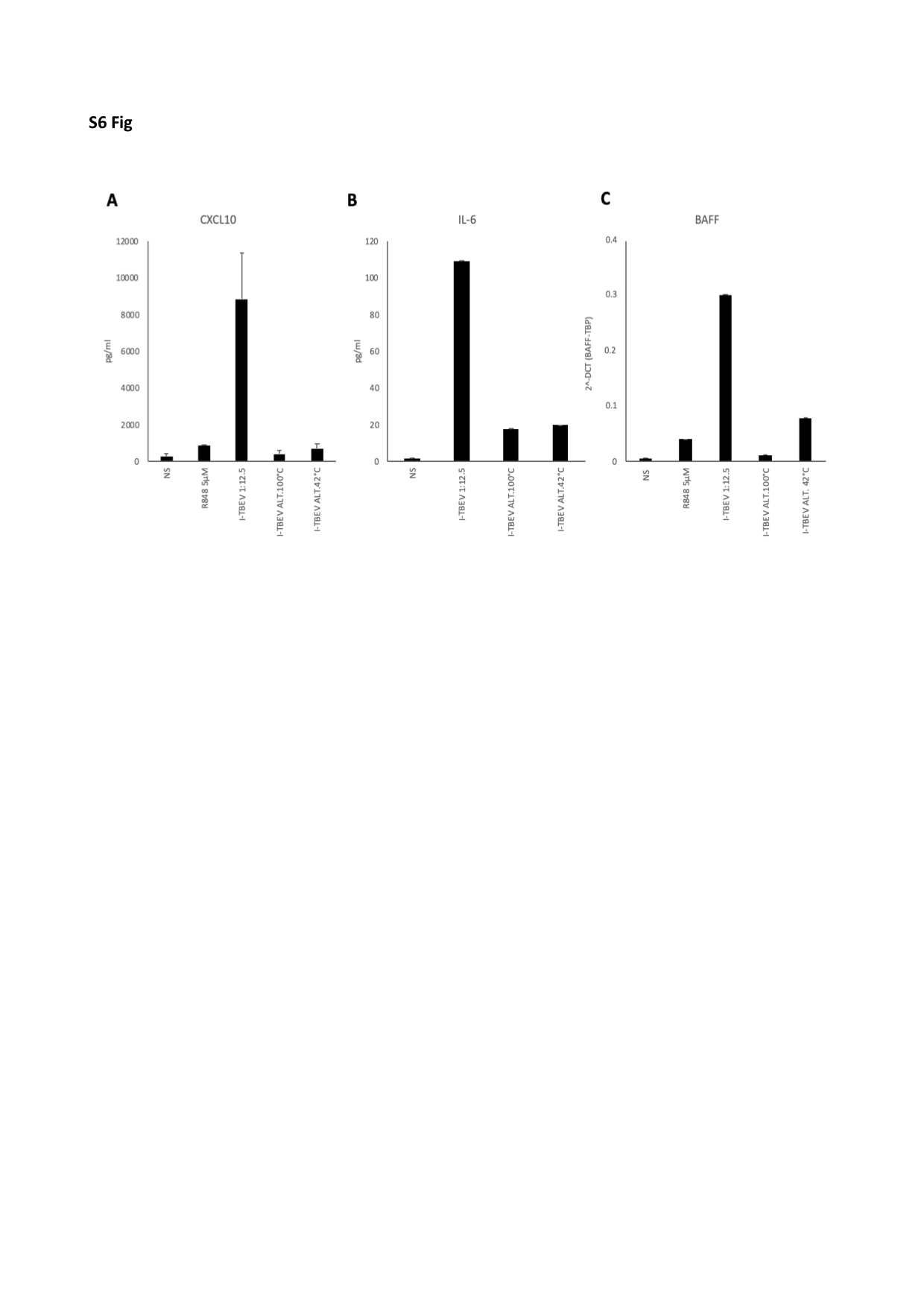

Supplement: S6 Fig — PBMC were left untreated (NS) or stimulated with R848 (5 μM), I-TBEV (dilution 1:12.5) and I-TBEV altered by temperature for 10 minutes at 100°C (I-TBEV ALT 100°C) or for 4 weeks at 42°C (I-TBEV ALT 42°C). (A-B) The production of CXCL10 and IL-6 was measured in culture supernatants collected after 24 hours by ELISA and cytometric bead assay, respectively. The results shown were mean values ± standard error of the mean (SEM) of 3 independent experiments. (C) Relative expression of BAFF was measured by q-PCR analysis on RNA extracted after 24 hours of stimulation. Data are normalized to TBP level by using the equation 2−ΔCt. The results shown were mean relative values ± SEM of 3 independent experiments. (TIFF) [file ppat.1009505.s006.tiff]

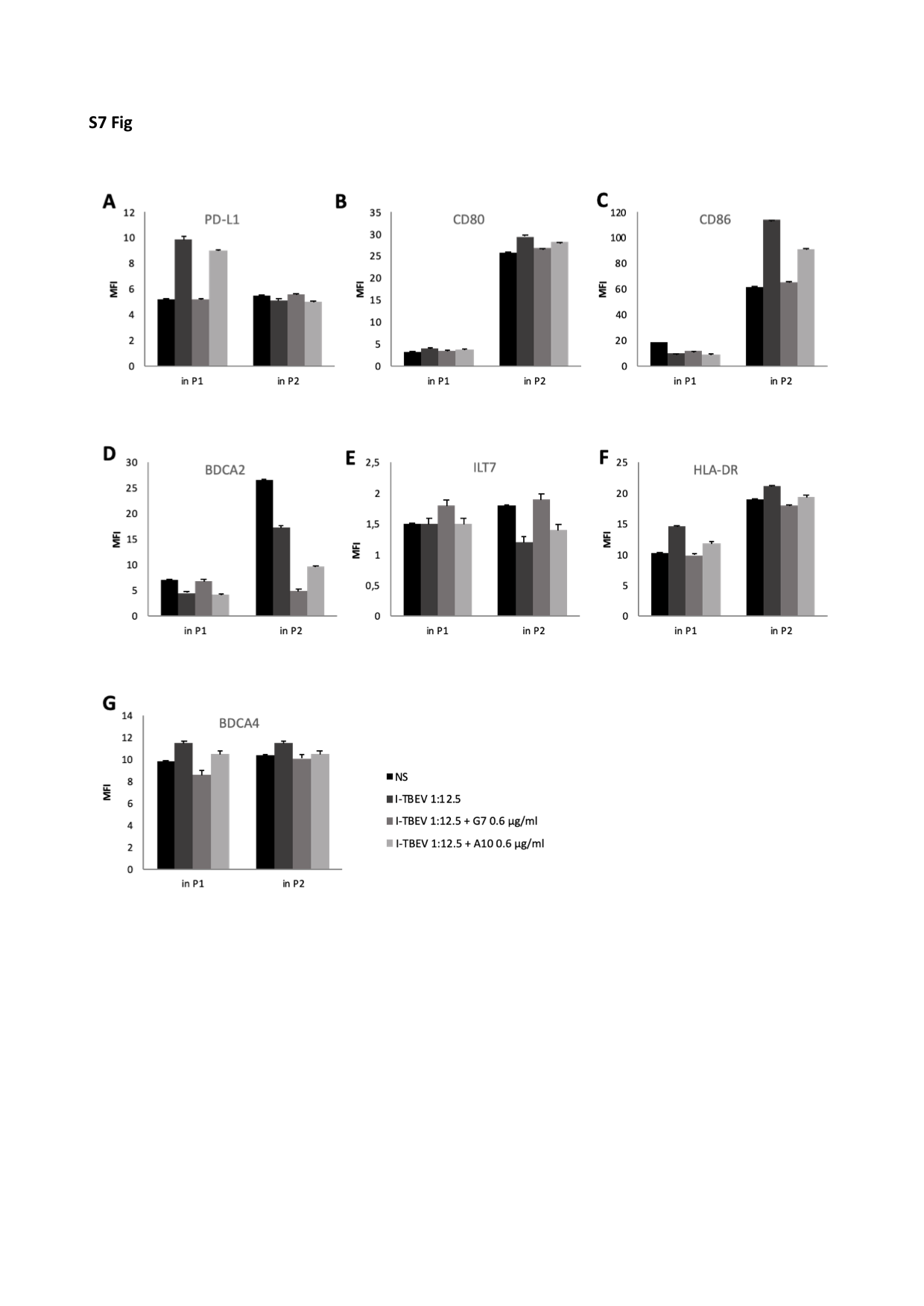

Supplement: S7 Fig — Isolated pDC were left untreated (NS) or stimulated for 24 hours with I-TBEV (dilution 1:12.5) alone or in combination either with single-chain antibody (scAb) blocking TBEV E glycoprotein, G7 (0.6 μg/ml) or with a non-related scAb, A10 clone (0.6 μg/ml). Two pDC sub-populations were characterized on the basis of PD-L1 and CD80 expression: P1-pDC (PD-L1+CD80–); P2-pDC (PD-L1+CD80+). The mean fluorescence intensity (MFI) of PD-L1, CD80, CD86, BDCA2, ILT7, HLA-DR and BDCA4 was determined by flow cytometer analysis. The results shown are mean values ± standard error of the mean of 2 independent experiments. (TIFF) [file ppat.1009505.s007.tiff]

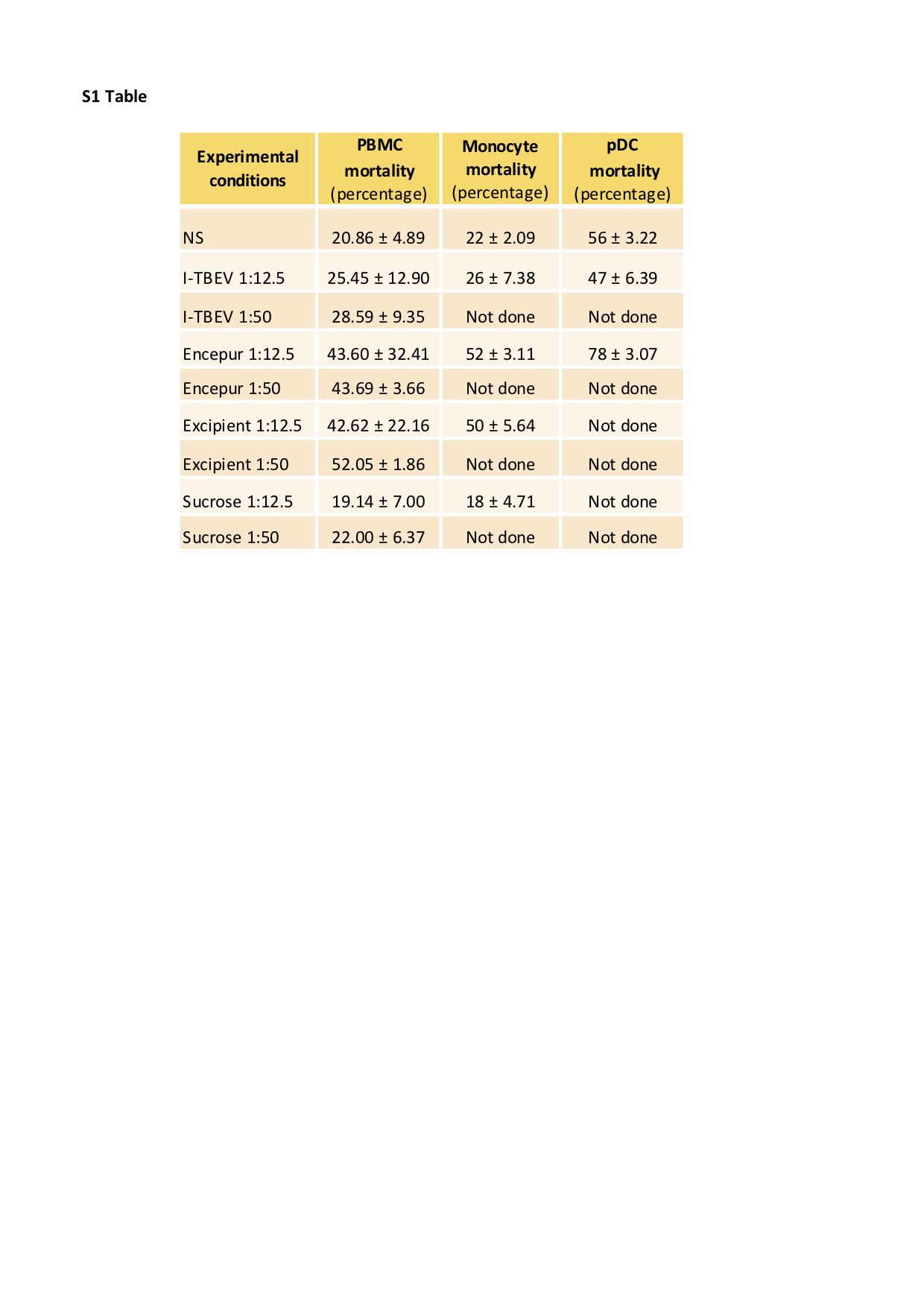

Supplement: S1 Table — Peripheral blood mononuclear cells (PBMC) were left untreated (NS) or stimulated with inactivated TBEV (I-TBEV), Encepur vaccine, excipient and sucrose (1:12.5 and 1:50) for 24 hours. Isolated monocytes or plasmacytoid dendritic cells (pDC) were left untreated (NS) or stimulated with inactivated TBEV (I-TBEV), Encepur vaccine, excipient and sucrose (1:12.5) for 24 hours. Cell viability was assessed by staining PBMC with the Fixable viability Dye (FvDye). The results shown are mean values ± standard error of the mean of 3 independent experiments for PBMC and mean values ± standard error of the mean of 2 independent experiments for monocytes and pDC. (TIFF) [file ppat.1009505.s008.tiff]

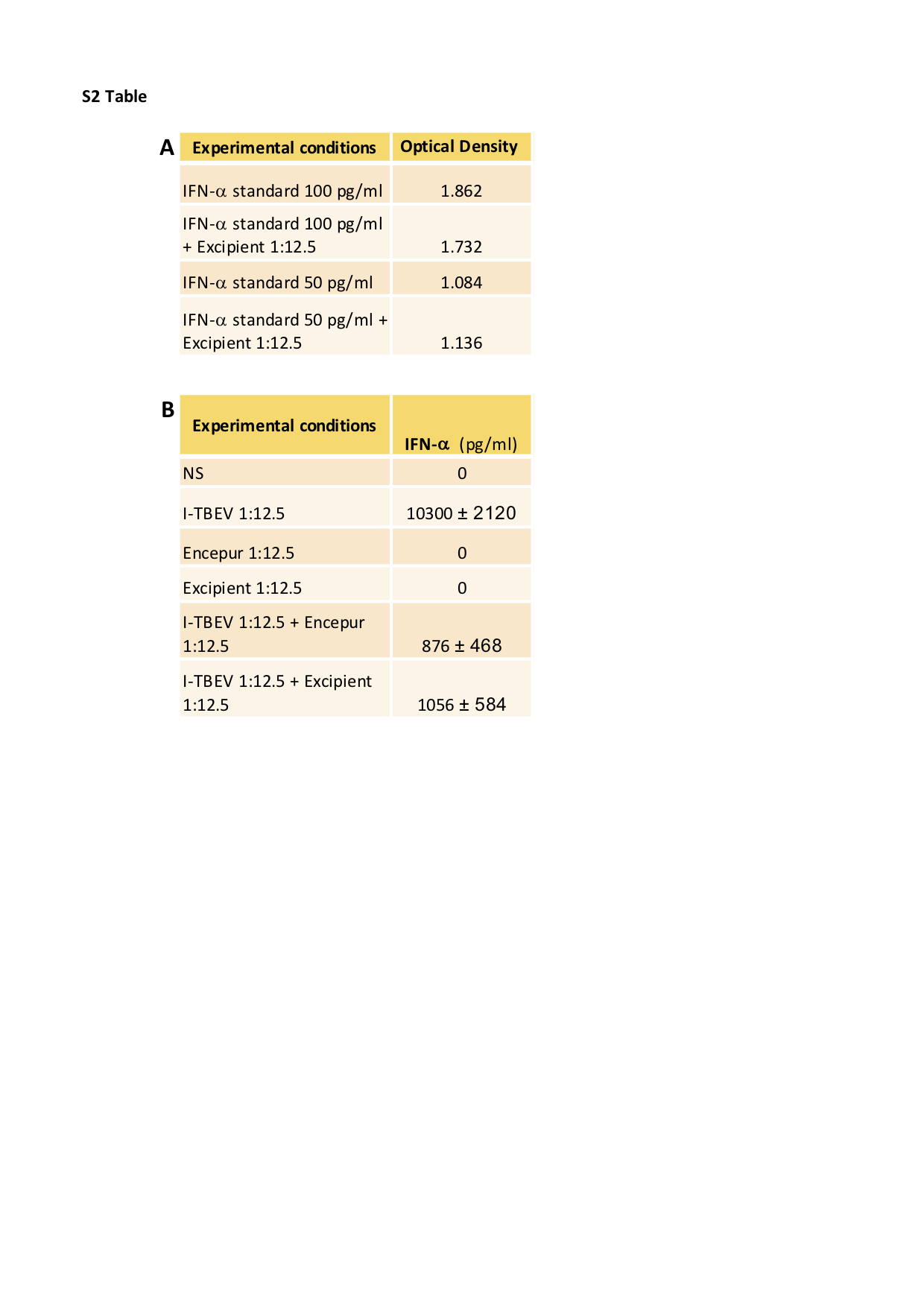

Supplement: S2 Table — (A) Technical interference of aluminum hydroxide was evaluated by adding Excipient (1:12.5) to IFN-α standard protein and then measuring the optical density value by ELISA. (B) Peripheral blood mononuclear cells (PBMC) were left untreated (NS) or stimulated with Encepur vaccine, excipient and inactivated TBEV (I-TBEV) alone or in combination either with Encepur vaccine or with excipient (1:12.5) for 24 hours. The production of IFN-α was measured in culture supernatants by ELISA. The results shown are mean values ± standard error of the mean of 3 independent experiments. (TIFF) [file ppat.1009505.s009.tiff]
